# Supplementary material for: Multimodal prehabilitation (Fit4Surgery) in high-impact surgery to enhance surgical outcomes: Study protocol of F4S PREHAB, a single center stepped wedge trial
Source: PLoS One. 2024 Jul 5;19(7):e0303829. doi: 10.1371/journal.pone.0303829 (PMC11226070; doi:10.1371/journal.pone.0303829)
Supplement: S1 Checklist — (DOC) [file pone.0303829.s001.doc]

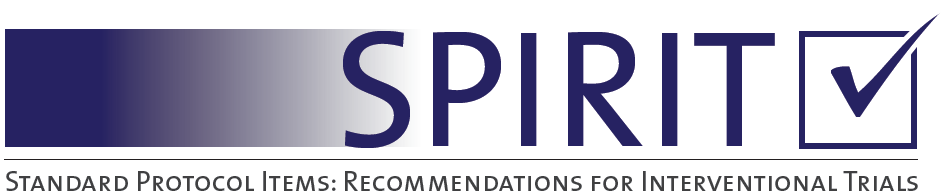


SPIRIT 2013 Checklist: Recommended items to address in a clinical trial protocol and related documents*

| Section/item | ItemNo | Description |
| --- | --- | --- |
| **Administrative information** | | |
| Title | 1 | Multimodal Prehabilitation (Fit4Surgery) in High-impact Surgery to Enhance Surgical Outcomes: Study Protocol of F4S PREHAB, a Single Center Stepped Wedge Trial |
| Trial registration | 2a | Netherlands Trial Register (NL8699). F4S PREHAB trial. CCMO: NL73777.091.20. |
|  | 2b | <https://trialsearch.who.int/Trial2.aspx?TrialID=NL8699> |
| Protocol version | 3 | 15 November 2020, version 9. |
| Funding | 4 | This is an investigator initiated trial. Whey protein supplements were funded by FrieslandCampina, Wageningen, the Netherlands. |
| Roles and responsibilities | 5a | D. Strijker, Radboudumc, coordinating investigator. Data collection, management, writing.  L.D. Drager, Radboudumc, coordinating investigator. Data collection, management, analysis, interpretation of data, writing.  M. van Asseldonk, Radboudumc, dietician. Study design.  F. Atsma, Radboudumc, methodologist. Study design, analysis.  M. van den Berg, Radboudumc, dietician. Study design.  E. van Daal, Radboudumc, epidemiologist. Study design, analysis.  L. van Heusden-Scholtalbers, Radboudumc, physiotherapist. Study design.  J. Meijerink, Radboudumc, surgeon and professor of innovative surgery. Study design.  P. Servaes, Radboudumc,  S. Teerenstra, Radboudumc, psychologist. Study design.  S. Verlaan, senior researcher. Study design, supervision.  B. van den Heuvel, Radboudumc, surgeon. Study design, supervision.  K. van Laarhoven, Radboudumc, head of department of Surgery and principal investigator. Study design, supervision. |
|  | 5b | Radboudumc Executive Board  P.O. Box 9101, 6500 HB Nijmegen  The Netherlands  Internal postal code 630  Geert Grooteplein Zuid 10  Radboudumc main entrance, route 630 T  +31 24 361 89 33 |
|  | 5c | Role of study sponsor and funders: none. |
|  | 5d | Not applicable. |
| Introduction |  |  |
| Background and rationale | 6a/b | High impact surgery has major consequences on the quality of life of patients. It takes them off normal work and life for prolonged periods and often they do not return to the preoperative level of daily activities and societal and work participation. Besides the regular impact of surgery, postoperative complications occur in up to 15-60% of patients and are associated with a higher mortality rate. The number and severity of complications is principally related to the initial quality of the surgical treatment. But also, it is strongly related to patient’s individual pre-operative functional capacity, pre-operative physical fitness, nutritional status, mental health, immune status and intoxications like alcohol abuse and smoking. Complex operations are the core business of academic surgical departments and the whole peri-operative process (surgery, anesthesiology, intensive care treatment), as post-operative morbidity and handling of complications is intensive and costly. Traditional approaches have mainly focused on minimizing operative trauma (minimal invasive operations) and peri-operative clinical recovery programs, such as the Enhanced Recovery After Surgery (ERAS) protocol. Recent evidence, however, shows that the preoperative period might be the optimal time frame for intervention to achieve short term and long lasting effects. During the last years, various so-called prehabilitation programs have been initiated with promising results. Prehabilitation, the optimization of a patient preoperatively, seems to prevent postoperative complications, enhance recovery after surgery and reduce cost of the burden of care. Therefore prehabilitation promises to be a straightforward intervention with a clear positive intervention-outcome correlation from both patient’s perspective and hospital’s perspective. However, high levels of evidence lack, due to poor methodology and lack of a comprehensive approach of previous studies on the effect of prehabilitation. Moreover, the mechanistical effects of prehabilitation have not been explained so far and the effects on a macro-economic level are not clear. |
|  |  | |
| Objectives | 7 | A stepped wedge trial with a clear aim to demonstrate the effects on clinical outcomes and the cost efficiency of prehabilitation in a hospital-wide setting. |
| Trial design | 8 | Stepped wedge design. |
| Methods: Participants, interventions, and outcomes | | |
| Study setting | 9 | Academic hospital. Radboudumc, Nijmegen, the Netherlands. |
| Eligibility criteria | 10 | Inclusion: all patients aged sixteen years and older undergoing elective high-impact surgery within 20 health clusters (including: colon cancer, rectal cancer, liver cancer or metastases (of colorectal origin), (retro)peritoneal malignancies, esophageal cancer, pancreaticobiliary cancer, abdominal aortic aneurysm (open and endovascular repair), oral cancer, laryngeal cancer, supratentorial meningioma, autologous breast reconstruction, endometrial cancer, ovarian cancer, vulvar cancer, hip arthrosis, hip or knee arthroplasty failure, renal cancer, and bladder cancer).  Exclusion criteria: chronic kidney disease stage ≥4 which contraindicates protein supplementation, cognitive disabilities or illiteracy (inability to read and understand the Dutch language), and characteristics that contraindicate or impede high-intensity exercise such as impaired mobility, premorbid conditions like cardiac or respiratory diseases, and ASA scores of ≥4. |
| Intervention | 11a/b | Patients participating in the control group will receive standard preoperative care in accordance with Dutch guidelines.  Patients assigned to the intervention group will undergo a multimodal prehabilitation program, consisting of: an exercise program, a nutritional intervention, psychological support, and smoking and alcohol cessation.  Exercise program:  - Endurance training (HIIT): interval training with a total duration of 28 minutes, preceded by a 2-minute warm-up, consisting of alternating intervals of high intensity (4 intervals of 4 minutes) and moderate intensity (4 intervals of 3 minutes). The high intensity workload will be set at 90% of the peak wattage achieved in the Steep Ramp Test, corresponding to an estimated 90% VO2 peak. The moderate intensity workload will be set at 30% of the peak wattage. Examples of aerobic exercise machines suitable for performing HIIT include a bicycle, rower, treadmill, and cross-trainer. The workload should be adjusted by 5-10% if a patient is unable to complete the high intensity intervals.  - Resistance training: this training targets all major muscle groups and comprises six exercises: leg press, chest press, abdominal crunch, low row, lat pulldown, and step up. Each exercise consists of two sets of 10 repetitions. The strength exercises will follow a pattern of two seconds of concentric strength and two seconds of eccentric strength. The weight for each exercise will be adjusted based on the indirect 1RM measured at baseline (25). The weight for the exercises will start at 65% of the calculated 1RM, with a weekly increase of 5% resulting in 80% of baseline 1RM by the fourth week of the exercise program. The weight should be adjusted by 5-10% based on a patient’s ability to complete 10 repetitions in the second set.  - Unsupervised training: patients will be instructed to engage in at least 60 minutes of aerobic exercise on days without supervised training. If physical capacity is insufficient, this can be divided into two or three periods of 20-30 minutes. Examples of aerobic exercises include walking, cycling, and swimming.  Nutritional intervention: all patients will be referred to a registered in-hospital dietician who will provide personalized dietary advice to optimize nutritional intake, focusing on protein, energy, and micronutrients. This guidance aims to achieve an anabolic state and to enhance the effects of physical training on lean body mass increment. Since protein intake is crucial to stimulate muscle protein synthesis, the goal is to achieve a daily protein intake of ≥1.5 g/kg/BW and a minimal intake of ≥1.2 g/kg/BW. To accomplish this, patients will receive high-quality whey protein shakes (Nutri Whey^TM^ Isolate, FrieslandCampina) containing 30 g of whey protein and 20 µg vitamin D to be consumed as one dose daily and an additional dose following supervised training (within one hour). Patients will be advised to distribute their dietary protein consumption evenly across meals, aiming for at least two meals per day containing 25 g protein or more.  To address potential vitamin deficiencies all patients will be provided with daily multivitamin supplementation, equivalent to 50% of the recommended daily intake. |
|  |  | Psychological support: to assess patients’ symptoms of anxiety and depression the Hospital Anxiety and Depression Scale (HADS) will be applied at baseline. Patients with scores ≥15 on the HADS will be referred to a trained psychologist who will provide support to optimize their psychological well-being and teach coping mechanisms specifically tailored to the surgical treatment. Patients with scores lower than 15 and a history of psychological health issues will be referred to a social worker. Additional sessions will be scheduled during the preoperative period as deemed necessary.  Smoking and alcohol cessation: all patients who are active smokers during the baseline assessment will be offered a comprehensive smoking cessation program, which includes counseling and nicotine replacement therapy, prior to surgery. Furthermore, all patients are advised to completely quit the consumption of alcoholic beverages. |
|  | 11c | Strategies to improve adherence:  - In between evaluation with participants by phone  Strategies to monitor adherence:  - Feedback form provided by supervising physiotherapist  - Questionnaires (patient-reported outcomes) |
|  | 11d | Planned scientific research, possibly interfering with multimodal prehabilitation (such as interventions aimed for lifestyle changes) are prohibited. |
| Outcomes | 12 | Primary outcome: occurrence and severity of postoperative complications, as measured by the Clavien-Dindo classification and Comprehensive Complication Index (CCI)  Secondary outcomes: length of hospital stay.  Tertiary outcomes: functional capacity (estimated VO_2_ peak, indirect 1RM, handgrip strength, Timed Chair Stand test, SQUASH questionnaire), nutritional status (length, BW, fat-free mass, PG-SGA questionnaire), mental health status (SF-36 and EQ-5D-5L questionnaires), intoxications (Health Behavior Questionnaire), and medical consumption (iMedical Consumption Questionnaire). |
| Participant timeline | 13 | See **Table 1 (p. 11)**. |
| Sample size | 14 | The sample size calculation is based on the assumption of a relative reduction of 20% in complication rates (Clavien-Dindo II or higher). To determine the required sample size, simulations were conducted using a log-binomial model with fixed effects of cluster (specific diagnosis), intervention, and time trend in absence of intervention (as a continuous variable). Monthly binary data were generated for each cluster per month, assuming a binary distribution that correlates with the estimated number of surgical procedures. This incorporated a modest overall time trend and the estimated complication rates for the control periods, while anticipating a 20% reduction in complication rates during the intervention periods (Table 1). Based on simulations, the power calculated was 83%. The anticipated total prospective patient inclusion is estimated to be 2828 patients. The prospective cohort will be complemented by a historical cohort. |
| Recruitment | 15 | Adequate participant enrolment will be achieved by the nature of the study design (stepped wedge). |
| **Methods: Assignment of interventions (for controlled trials)** | | |
| Allocation: |  |  |
| Sequence generation | 16a | Not applicable. |
| Allocation concealment mechanism | 16b | Not applicable. |
| Implementation | 16c | Not applicable. |
| Blinding (masking) | 17a | Not applicable. |
|  | 17b | Not applicable. |
| **Methods: Data collection, management, and analysis** | | |
| Data collection methods | 18a | Plans for assessment and collection of outcome, baseline, and other trial data: all participants are screened at baseline and relevant parameters are directly imported into Castor EDC. Primary outcomes are collected 30 days after surgery and will be documented in Castor EDC. Questionnaires following at 3, 6, and 12 months will be automatically emailed to corresponding participants. |
|  | 18b | Plans to promote participant retention and complete follow-up: participants will receive a standard appointment 3 months after surgery alongside an outpatient clinic appointment. Patients are reminded by notifications at 6 and 12 months follow-up regarding unfinished questionnaires. |
| Data management | 19 | Study data, including information retrieved from measurements, patient records and questionnaires, will be collected and stored in Castor EDC. Every patient will receive a unique patient study number (i.e. F4S_0001, F4S_0002 et cetera). Paper files like informed consent and questionnaires will be stored in a locked cabinet at the department of Surgery, in the Radboudumc.  Data will be coded by using the unique patient study numbers and a codelist will be produced and electronically stored an locked on the department’s internal drive. The codelist will be freezed and printed at the end of the study, on which a name, signature and date will be noted.  Radboudumc’s Digital Research Environment (DRE) will be used for archiving data and analysis files. The print of the codelist will be scanned and archived on the department’s internal drive. Study data will be saved for 15 years after termination of the study. Using study data in future research after this term is only possible after renewed permission by the patient as stated in the informed consent. |
| Statistical methods | 20a/b/c | Baseline descriptive characteristics of the patient cohort will be described as mean and standard deviation (SD) for continuous variables, and count and percentage for categorical variables. The effect of the intervention on the primary outcome, the occurrence of Clavien-Dindo grade II complications or higher, will be analyzed using a log-binomial model (generalized linear model with log-link and binomial error distribution) fitted with clusters (categorical variable), treatment arm, and time (as linear continuous) as fixed effects. Fixed effects for clusters are chosen as the aim is primarily to establish the effect for the Radboudumc. Additionally, both Clavien-Dindo complications and CCI scores will be analyzed as semi-continuous and continuous scores, respectively, using generalized linear models with log-link, normal distribution for the errors, and the same fixed effects. Moreover, other parametrizations may also be explored if these improve the model fit, such as random effects for clusters. Primary analyses will be performed according to an intention-to-treat as well as a per-protocol approach.  Secondary and tertiary outcomes will be analyzed using regression models. Linear regression will be used for continuous outcome measures, and logistic regression for binary outcomes. Moreover, potential differences in outcomes between control and intervention group will be tested per single time point. Independent samples t-tests and Mann-Whitney U tests will be applied to analyze normally and non-normally distributed continuous parameters, whereas chi-square tests or logistic, ordinal or nominal regression models will be used to analyze categorical parameters. |
| **Methods: Monitoring** | | |
| Data monitoring | 21a | Composition of data monitoring committee (DMC): not applicable. |
|  | 21b | Description of any interim analyses: not applicable. Stopping guidelines: in case of significantly increased incidence of serious side-effects or adverse events in patients within the intervention group, the study will be prematurely terminated. |
| Harms | 22 | Plans for collecting, assessing, reporting, and managing solicited and spontaneously reported adverse events and other unintended effects of trial interventions or trial conduct: all serious adverse events will be reported through the web portal ‘ToetsingOnline’ to the accredited medical ethics committee that approved the protocol. |
| Auditing | 23 | Frequency and procedures for auditing trial conduct, if any, and whether the process will be independent from investigators and the sponsor: auditing is organized once per year and is independent from the investigators. |
| Ethics and dissemination | | |
| Research ethics approval | 24 | Plans for seeking research ethics committee/institutional review board (REC/IRB) approval: the protocol was approved by the local Medical Ethics Committee (METC Oost-Nederland) (NL73777.091.20). |
| Protocol amendments | 25 | Plans for communicating important protocol modifications: all intended and relevant modifications will be submitted to the relevant Medical Ethics Committee for review and approval. |
| Consent or assent | 26a | Who will obtain informed consent or assent from potential trial participants or authorised surrogates, and how: during the initial outpatient clinic visit, patients will be provided with detailed information regarding the study. Prior to participation, written informed consent will be obtained from all patients. Informed consent will be obtained by the coordinating investigators or authorized study personnel. |
|  | 26b | Additional consent provisions for collection and use of participant data and biological specimens in ancillary studies, if applicable: not applicable. |
| Confidentiality | 27 | How personal information about potential and enrolled participants will be collected, shared, and maintained in order to protect confidentiality before, during, and after the trial: see 19. |
| Declaration of interests | 28 | Financial and other competing interests for principal investigators for the overall trial and each study site: there are no declarations of interest to report. |
| Access to data | 29 | Statement of who will have access to the final trial dataset, and disclosure of contractual agreements that limit such access for investigators: the coordinating and supervising investigators will have access to the final trial dataset. |
| Ancillary and post-trial care | 30 | Provisions, if any, for ancillary and post-trial care, and for compensation to those who suffer harm from trial participation:  The sponsor/investigator has a liability insurance which is in accordance with article 7 of the WMO. The sponsor (also) has an insurance which is in accordance with the legal requirements in the Netherlands (Article 7 WMO). This insurance provides cover for damage to research subjects through injury or death caused by the study. The insurance applies to the damage that becomes apparent during the study or within 4 years after the end of the study. |
| Dissemination policy | 31a | Plans for investigators and sponsor to communicate trial results to participants, healthcare professionals, the public, and other relevant groups (eg, via publication, reporting in results databases, or other data sharing arrangements), including any publication restrictions: trial results will be reported via publication. |
|  | 31b | Authorship eligibility guidelines and any intended use of professional writers: authorship is determined and there will be no use of professional writers. |
|  | 31c | Plans, if any, for granting public access to the full protocol, participant-level dataset, and statistical code: the full protocol, participant-level dataset, and statistical code will be accessible on request. |
| Appendices |  |  |
| Informed consent materials | 32 | See **Appendix 1** for informed consent (p. 12) and patient information form (p. 14 onwards)**.** |
| Biological specimens | 33 | Not applicable. |

*It is strongly recommended that this checklist be read in conjunction with the SPIRIT 2013 Explanation & Elaboration for important clarification on the items. Amendments to the protocol should be tracked and dated. The SPIRIT checklist is copyrighted by the SPIRIT Group under the Creative Commons “[Attribution-NonCommercial-NoDerivs 3.0 Unported](http://www.creativecommons.org/licenses/by-nc-nd/3.0/)” license.

**Table 1. Overview of preoperative, perioperative, and postoperative measurements.**

| **Baseline** | **After prehabilitation and prior to surgery** | **Perioperative period** | **+3 months** | **+6 months** | **+12 months** |
| --- | --- | --- | --- | --- | --- |
| *Functional capacity and activity*   - VO_2_ peak (Steep Ramp Test) - Indirect 1 repetition measures (1RM) - Hand grip strength - Timed Chair Stand test (5-CST) - Short Questionnaire to Assess Health-enhancing physical activity (SQUASH) | *Functional capacity and activity*   - VO_2_ peak (Steep Ramp Test) - Indirect 1 repetition measures (1RM) - Hand grip strength - Timed Chair Stand test (5-CST) | *Postoperative complications*   - Clavien-Dindo classification - Comprehensive Complication Index (CCI) | *Functional capacity and activity*   - VO_2_ peak (Steep Ramp Test) - Indirect 1 repetition measures (1RM) - Hand grip strength - Timed Chair Stand test (5-CST)   Short Questionnaire to Assess Health-enhancing physical activity (SQUASH) | *Functional activity*   - Short Questionnaire to Assess Health-enhancing physical activity (SQUASH) | *Functional activity*   - Short Questionnaire to Assess Health-enhancing physical activity (SQUASH) |
| *Nutritional status*   - Length - Body weight (BW) - Fat-free mass (FFM) - PG-SGA SF questionnaire - 3-day food diary | *Nutritional status*   - Length - Body weight (BW) - Fat-free mass (FFM) - PG-SGA SF questionnaire - 3-day food diary | *Length of hospital stay (LoS)* | *Nutritional status*   - Length - Body weight (BW) - Fat-free mass (FFM) - PG-SGA SF questionnaire | *Nutritional status*  N/A | *Nutritional status*  N/A |
| *Mental health*   - SF-36 questionnaire - Hospital Anxiety and Depression Scale (HADS) | *Mental health*   - SF-36 questionnaire |  | *Mental health*   - SF-36 questionnaire | *Mental health*   - SF-36 questionnaire - EuroQuol 5D (EQ-5D-5L) | *Mental health*   - SF-36 questionnaire |
| *Medical consumption*   - N/A | *Medical consumption*   - iMedical Consumption Questionnaire (iMCQ) |  | *Medical consumption*   - iMedical Consumption Questionnaire (iMCQ) | *Medical consumption*   - iMedical Consumption Questionnaire (iMCQ) | *Medical consumption*   - iMedical Consumption Questionnaire (iMCQ) |
| *Health behavior*   - N/A | *Health behavior*   - Health behavior questionnaire |  | *Health behavior*   - Health behavior questionnaire |  |  |

**Appendix 1. Informed consent and patient information form**

# Fit4Surgery: prehabilitatie voorafgaand aan zware operaties

- Ik heb de informatiebrief gelezen. Ook kon ik vragen stellen. Mijn vragen zijn voldoende beantwoord. Ik had genoeg tijd om te beslissen of ik meedoe.
- Ik weet dat meedoen vrijwillig is. Ook weet ik dat ik op ieder moment kan beslissen om toch niet mee te doen of te stoppen met het onderzoek. Daarvoor hoef ik geen reden te geven.
- Ik geef toestemming voor het verzamelen en gebruiken van mijn gegevens voor de beantwoording van de onderzoeksvraag in dit onderzoek

- Ik weet dat voor de controle van het onderzoek sommige mensen toegang tot al mijn gegevens kunnen krijgen. Die mensen staan vermeld in deze informatiebrief. Ik geef toestemming voor die inzage door deze personen.

- Ik geef toestemming voor het informeren van mijn behandelend specialist van onverwachte bevindingen die van belang (kunnen) zijn voor mijn gezondheid
- Ik geef □ **wel**

# geen

toestemming om mijn persoonsgegevens langer te bewaren en te gebruiken voor toekomstig onderzoek op het gebied van prehabilitatie voorafgaand aan zware operaties

- Ik geef □ **wel**

# geen

toestemming om mij na dit onderzoek opnieuw te benaderen voor een vervolgonderzoek

- Ik wil meedoen aan dit onderzoek

Naam proefpersoon:

Handtekening: Datum : / /

-----------------------------------------------------------------------------------------------------------------

Ik verklaar dat ik deze proefpersoon volledig heb geïnformeerd over het genoemde onderzoek.

Als er tijdens het onderzoek informatie bekend wordt die de toestemming van de proefpersoon zou kunnen beïnvloeden, dan breng ik hem/haar daarvan tijdig op de hoogte.

Naam onderzoeker (of diens vertegenwoordiger):

Handtekening: Datum: / /

------------------------------------------------------------------------------------------------------------------

Aanvullende informatie is gegeven door: Naam:

Functie:

Handtekening: Datum: / /

------------------------------------------------------------------------------------------------------------------

* Doorhalen wat niet van toepassing is

Patiënten informatie folder “Fit4Surgery: prehabilitatie bij zware operaties”

Geachte heer/mevrouw,

Uw chirurg, case manager of verpleegkundig specialist heeft met u besproken dat u op korte termijn wordt geopereerd. Graag informeren wij u over het medisch‐wetenschappelijk onderzoek van het Radboud universiteit medisch centrum in Nijmegen naar het effect van een prehabilitatie programma voorafgaand aan zware operaties.

U beslist zelf of u wilt meedoen aan het medisch‐wetenschappelijk onderzoek. Voordat u deze beslissing kunt nemen, is het belangrijk dat u goed bent geïnformeerd. Wij adviseren u om deze informatie folder rustig door te lezen. Voor vragen kunt u de arts‐onderzoeker of onafhankelijke deskundige, die aan het eind van deze brief wordt genoemd, om informatie vragen. Verdere algemene informatie over het meedoen aan wetenschappelijk onderzoek kunt u vinden op de website van de Rijksoverheid: [www.rijksoverheid.nl/mensenonderzoek](http://www.rijksoverheid.nl/mensenonderzoek).

## Algemene informatie

Dit onderzoek wordt uitgevoerd door de onderzoekers van de afdelingen Heelkunde, Urologie, Orthopedie, Neurochirurgie, Plastische chirurgie, Hoofd‐hals chirurgie, Gynaecologie en Cardio‐ thoracale chirurgie van het Radboud universiteit medisch centrum. De medisch‐ethische toetsingscommissie CMO Arnhem‐Nijmegen heeft dit onderzoek goedgekeurd. Informatie over het toetsingsprocedure voor wetenschappelijk onderzoek vindt u op de website van de Rijksoverheid.

## Het doel van het onderzoek

Het doel van dit onderzoek is om vast te stellen wat het effect is van prehabilitatie (het verbeteren van de conditie voorafgaand aan een operatie) op het herstel na een zware operatie.

## 3. Achtergrond van het onderzoek

## Hoewel de technieken van operaties in de afgelopen tijd steeds beter zijn geworden, kent deze behandeling ook serieuze bijwerkingen, zoals wondinfecties, bloedingen of heroperaties. Het herstel duurt soms erg lang en sommige mensen komen nooit meer terug op hun oude functioneringsniveau. Om het risico op bijwerkingen te verkleinen, is het belangrijk dat u een zo goed mogelijke conditie heeft rondom de operatie.

Prehabilitatie is het verbeteren van de conditie voorafgaand aan een operatie. Kanker of een andere zware diagnose en een operatie vergen veel van uw lichaam. Met een goede conditie bent u beter in staat om de operatie goed te doorstaan, is de kans op complicaties kleiner en gaat het herstel vlotter.

Uit een testonderzoek (pilotstudie) in het Radboud universitair medisch centrum is gebleken dat darmkanker patiënten die een prehabilitatie programma hebben ondergaan een kleinere kans hebben op complicaties en sneller herstellen na een operatie. In dit onderzoek willen wij het effect van prehabilitatie op het herstel na een zware operaties vaststellen. Dit doen we door het herstel na een zware operatie te vergelijken tussen patiënten die normale zorg ondergaan en patiënten die een prehabilitatie programma ondergaan.

## 4. Wat meedoen inhoudt

In dit onderzoek zullen de deelnemende patiënten die een zware operatie ondergaan worden ingedeeld in twee verschillende groepen: groep A zal de normale zorg voorafgaand aan de operatie krijgen, groep B zal een prehabilitatie programma ondergaan. Loting bepaald in welke groep u wordt ingedeeld.

**Bezoeken en metingen**

Ongeacht in welke groep u wordt ingedeeld, nodigen wij u tijdens dit onderzoek meerdere keren uit in het ziekenhuis voor verschillende testen. Ook vragen wij u op bepaalde tijdstippen voor en na de operatie online vragenlijsten in te vullen. Een overzicht hiervan vindt u in onderstaande tabel.

| Tijdstip onderzoek |  |
| --- | --- |
| Bij start van het programma | Bezoek bij de onderzoeker  Vragenlijsten over uw activiteiten en kwaliteit van leven |
| Week van de operatie | Bezoek bij de onderzoeker  Vragenlijsten over uw activiteiten en kwaliteit van leven |
| 6 maanden na de operatie | Vragenlijsten over uw activiteiten en kwaliteit van leven (via mail) |
| 1 jaar na de operatie | Vragenlijsten over uw activiteiten en kwaliteit van leven (via mail) |

Tijdens het bezoek bij de onderzoeker zult u een inspanningstest op de fiets ondergaan, terwijl wij de inspanning volgen met een hartslagmeter. Op deze manier krijgen wij een goed beeld van uw fitheid. Ook zal de onderzoeker tijdens uw bezoek aan de hand van verschillende oefeningen met gewichten uw maximale kracht bepalen. Tevens zal uw lichaamssamenstelling worden bepaald door het meten van uw lengte en gewicht. Ook zullen uw vetpercentage opmeten.

De vragenlijsten worden u online toegestuurd en zijn bedoeld om uw activiteiten en kwaliteit van leven in kaart te brengen.

**Zorg voorafgaand aan de operatie**

Indien u wordt ingedeeld in groep A, ondergaat u de zorg voorafgaand aan de operatie zoals die normaal gesproken wordt aangeboden in het Radboud universitair medisch centrum.

Indien u wordt ingedeeld in groep B, ondergaat u een speciaal ontworpen prehabilitatie programma. Het programma vindt plaats in de weken voorafgaand aan uw operatie en bestaat uit 4 onderdelen:

1. Gezonde voeding
2. Fysieke training
3. Mentale ondersteuning
4. Stoppen met roken programma


   1. *Gezonde voeding:*

Eten en drinken kan door kanker of een andere zware diagnose lastig zijn. Wanneer u te veel afvalt, kunt u ondervoed raken. Dat is niet goed voor het verdragen van de operatie en het herstel erna. Een diëtist zal u begeleiden zodat u in een zo’n goed mogelijke voedingstoestand de operatie in zal gaan. Dit bestaat uit:

- Uw voedingsstatus bekijken
- Een persoonlijke plan om uw voeding te optimaliseren
- Een aanvulling van extra eiwitten en vitaminen, om eventuele tekorten aan te vullen en de spieropbouw te bevorderen
  1. *Fysieke training*

Onder begeleiding van een fysiotherapeut in de buurt zult u aan uw lichamelijke conditie gaan werken. Dit programma zal er als volgt uitzien:

- 2x of 3x per week onder begeleiding van een fysiotherapeut krachttraining en intervaltraining. De invulling van de training wordt speciaal op u afgestemd
- 4x of 5x per week 60 minuten per dag wandelen of fietsen. Dit is op de dagen dat u niet in het ziekenhuis bent voor de training onder begeleiding van de fysiotherapeut. Dit bewegingsadvies mag verdeeld worden over 2‐3 verschillende momenten van 10‐20 minuten bewegen. Met de extra eiwitten en vitamines, gegeven door een diëtist in het ziekenhuis, wordt het effect van deze trainingen versterkt
- Daarnaast zal de fysiotherapeut u ontspanningsoefeningen aanreiken. U bepaalt zelf in hoeverre u hier thuis gebruik van maakt
  1. *Mentale ondersteuning*

Het ondergaan van een operatie is vaak spannend en kan angstige gevoelens oproepen. Deze gevoelens kunnen invloed hebben op uw sociale en functionele activiteiten, waardoor uw (mentale) gezondheid achteruit kan gaan. Wij begeleiden u graag gedurende deze lastige periode. Door middel van een vragenlijst zullen wij het risico op het krijgen van psychische klachten inschatten. Indien nodig zullen wij een afspraak bij een medisch psycholoog inplannen. Dit gaat altijd in overleg met u.

- 1. *Stoppen met roken programma*

Roken vergroot de kans op problemen na de operatie, zoals een bloeding of de vorming van bloedstolsels (trombose). Ook genezen wonden minder snel als u rookt en leidt het tot het langer aanhouden van ontstekingen. Onderzoek heeft aangetoond dat het stoppen met roken voor een operatie het aantal complicaties na een operatie verminderd. Mocht u normaal gesproken roken, dan bieden wij u een stopprogramma aan met intensieve begeleiding en nicotine vervangend therapie.

## Wat wordt er van u verwacht

Om het onderzoek goed te laten verlopen, is het belangrijk dat u zich aan de volgende afspraken houdt tijdens het onderzoek.

De afspraken zijn dat u:

‐ Afspraken van bezoeken voor de studie nakomt (groep A en B)

‐ Het prehabilitatie programma uitvoert volgens uitleg (groep B)

Het is belangrijk dat u contact opneemt met de onderzoeker:

‐ Als u in een ziekenhuis wordt opgenomen of behandeld

‐ Als u te horen heeft gekregen dat u eerder geopereerd wordt

‐ Als u plotseling gezondheidsklachten krijgt

‐ Als u niet meer wilt meedoen aan het onderzoek

‐ Als uw contactgegevens wijzigen

## Mogelijke ongemakken

Het risico van deelname aan dit onderzoek is verwaarloosbaar klein.

Deelnemers in groep A: u ondergaat de standaard zorg voorafgaand aan een operatie. Er is geen extra risico voor u bij deelname aan dit onderzoek.

Deelnemers in groep B: het prehabilitatie programma wordt aangepast op uw persoonlijke conditie, kracht en voedingstoestand. Daarbij voert u de interval‐ en krachttraining altijd onder begeleiding uit. Het risico van deelname aan dit onderzoek is daarom verwaarloosbaar klein. U kunt altijd met de fysiotherapeut overleggen over vragen of problemen die zich voordoen. Indien nodig, kan de fysiotherapeut in overeenstemming met u het prehabilitatie programma aanpassen gedurende het onderzoek.

Dit onderzoek gaat echter gepaard met enkele ongemakken, die hieronder puntsgewijs worden toegelicht:

‐ Overtraining of overbelasting: omdat u mogelijk meer gaat bewegen dan normaal, is er een kans dat u overtraind of overbelast raakt door het prehabilitatie programma. De fysiotherapeut zal u tijdens de trainingen goed in de gaten houden. Indien de fysiotherapeut aanwijzingen ziet van overtraining of overbelasting, zal hij of zij dit altijd met u bespreken. Indien u zich zelf zorgen maakt over overtraining of overbelasting, kunt u dit altijd aangeven bij de fysiotherapeut. U beslist samen over het vervolg van het prehabilitatie programma.

## Mogelijke voor‐ en nadelen

Het is belangrijk dat u de mogelijke voor‐ en nadelen goed afweegt voordat u besluit mee te doen aan dit onderzoek.

Voordelen

Deelnemers in groep A: u heeft zelf geen voordeel van meedoen aan dit onderzoek. Uw deelname kan wel bijdrage aan meer kennis over uw conditie en het effect van een prehabilitatie op het herstel na een zware operatie.

Deelnemers in groep B: uit een testonderzoek (pilotstudie) in het Radboud universitair medisch centrum is gebleken dat darmkanker patiënten die een prehabilitatie programma hebben ondergaan een kleinere kans hebben op complicaties en sneller herstellen na een operatie. Wij verwachten het zelfde effect in andere groepen patiënten die een zware operatie ondergaan.

Nadelen

Nadelen van meedoen aan het onderzoek kunnen zijn:

‐ Mogelijke ongemakken van de metingen in het onderzoek

Deelname aan het onderzoek betekent ook:

‐ Dat u extra tijd kwijt bent

‐ Extra testen

‐ Dat u afspraken heeft waaraan u zich moet houden Al deze zaken zijn hiervoor onder punt 4, 5 en 6 beschreven.

## Als u niet wilt meedoen of wilt stoppen met dit onderzoek

U beslist zelf of u meedoet aan dit onderzoek. Deelname is vrijwillig. Als u niet meedoet heeft dit geen enkel gevolg voor uw verdere behandeling. Indien u wel meedoet, behoudt u het recht om op ieder gewenst moment en zonder opgave van reden, uw deelname aan het programma te beëindigen. Ook dit zal geen enkel gevolg hebben voor uw normale verdere behandeling. U hoeft niet te zeggen waarom u stopt. Wel moet u dit direct melden aan de onderzoeker. De gegevens die tot dat moment zijn verzameld, zullen worden gebruikt voor onderzoek.

Als er nieuwe informatie over het onderzoek is die belangrijk voor is, laat de onderzoeker dit aan u weten. U wordt dan gevraagd of u blijft meedoen.

## Einde van het onderzoek

Uw deelname aan dit onderzoek stopt als:

‐ Alle bezoeken en metingen zoals beschreven bij punt 4 voorbij zijn

‐ U zelf kiest om te stoppen

‐ De onderzoeker, chirurg, sportarts, fysiotherapeut, diëtist of psycholoog het beter voor u vindt om te stoppen

‐ Het Radboud universitair medisch centrum, de overheid of de beoordeelde medisch‐ethische toetsingscommissie, besluit om het onderzoek te stoppen

Het hele onderzoek is afgelopen als alle deelnemers klaar zijn. Na het verwerken van alle gegevens informeert de onderzoeker u over de belangrijkste uitkomsten van het onderzoek. Dit gebeurt ongeveer 2 jaar na uw deelname.

## Gebruik en bewaren van uw gegevens

Voor dit onderzoek worden uw persoonsgegevens verzameld, gebruikt en bewaard. Het gaat om gegevens zoals uw naam, adres, geboortedatum en om gegevens over uw gezondheid rondom de operatie (zoals opnameduur en eventuele complicaties). Het verzamelen, gebruiken en bewaren van uw gegevens is nodig om de vragen die in dit onderzoek worden gesteld te kunnen beantwoorden en de resultaten te publiceren. Voor het gebruik van uw gegevens vragen wij om uw toestemming.

**Vertrouwelijkheid van uw gegevens**

Om uw privacy te beschermen krijgen uw gegevens een code. Uw naam en andere gegevens die u direct kunnen identificeren worden daarbij weggelaten. Alleen met de sleutel van de code zijn de gegevens tot u te herleiden. De sleutel van de code blijft veilig opgeborgen in het Radboud universitair medisch centrum. In rapporten en publicaties over het onderzoek zijn de gegevens niet tot u te herleiden.

**Toegang tot uw gegevens voor controle**

Sommige personen kunnen toegang krijgen tot al uw gegevens. Ook tot de gegevens zonder de code. Dit is nodig om te kunnen controleren of het onderzoek goed en betrouwbaar is uitgevoerd. Personen die ter controle inzage krijgen in uw gegevens zijn: de commissie die de veiligheid van het onderzoek in de gaten houdt, nationale en internationale toezichthoudende autoriteiten, bijvoorbeeld, de Inspectie Gezondheidszorg en Jeugd. Zij houden uw gegevens geheim. Wij vragen u voor deze inzage toestemming te geven.

**Bewaartermijn gegevens**

Uw gegevens worden 15 jaar bewaard in het Radboud universitair medisch centrum.

Bewaren en gebruik van gegevens voor ander onderzoek

Uw gegevens kunnen na afloop van dit onderzoek ook nog van belang zijn voor ander wetenschappelijk onderzoek. Daarvoor zullen uw gegevens 15 jaar worden bewaard. U kunt op het toestemmingsformulier aangeven of u hier wel of niet mee instemt. Indien u hier niet mee instemt, kunt u gewoon deelnemen aan dit onderzoek.

**Informatie over onverwachte bevindingen**

Tijdens dit onderzoek kan er per toeval iets gevonden worden dat niet van belang is voor dit onderzoek maar wel voor u. Als dit belangrijk is voor uw gezondheid, dan zult u op de hoogte worden gesteld door een arts. U kunt dan met uw arts bespreken wat er gedaan moet worden. Ook hiervoor geeft u toestemming.

**Intrekken toestemming**

U kunt uw toestemming voor gebruik van uw persoonsgegevens altijd weer intrekken. Dit geldt voor dit onderzoek en ook voor het bewaren en het eventuele gebruik voor het toekomstige onderzoek. De onderzoeksgegevens die zijn verzameld tot het moment dat u uw toestemming intrekt worden nog wel gebruikt voor het onderzoek.

**Meer informatie over uw rechten bij verwerking van gegevens**

Voor algemene informatie over uw rechten bij verwerking van uw persoonsgegevens kunt u de website van de Autoriteit Persoonsgegevens raadplegen. Bij vragen over uw rechten kunt u contact opnemen met de verantwoordelijke voor de verwerking van uw persoonsgegevens. Zie bijlage A voor alle contactgegevens. Bij vragen of klachten over de verwerking van uw persoonsgegevens raden we u aan eerst contact op te nemen met het Radboud universiteit medisch centrum. U kunt ook contact opnemen met de Functionaris voor Gegevens‐bescherming van het Radboud universitair medisch centrum (contactinformatie in bijlage A) of de Autoriteit Persoonsgegevens.

## Verzekering voor proefpersonen

Voor alle deelnemers die aan dit onderzoek meedoen, is een verzekering afgesloten. De verzekering dekt schade door het onderzoek. Niet alle schade is gedekt. In bijlage B vindt u meer informatie over de verzekering en de uitzonderingen. Daar staat ook aan wie u schade kunt melden.

## Vergoeding voor meedoen

De kosten die gepaard gaan met de metingen en het prehabilitatie programma worden niet bij u in rekening gebracht. Omdat u als deelnemer van dit onderzoek vaker naar het Radboud universitair medisch centrum zult komen voor metingen en/of trainingen, maakt u mogelijk extra reiskosten. De reiskosten van de extra bezoeken (dus niet de standaard bezoeken aan het Radboud universitair medisch centrum rondom een operatie) worden vergoed tot 10 euro per deelnemer per bezoek.

## Heeft u vragen?

Mocht u vragen hebben over het onderzoek of uw rechten als deelnemer aan het onderzoek dan kunt u het beste contact opnemen met uw casemanager, uw behandelend chirurg of arts‐onderzoeker. Als u liever een onafhankelijk persoon spreekt, kunt u terecht bij prof. dr. Niels. P. Riksen. Contactinformatie vindt u in bijlage A.

## Wilt u meedoen?

Wanneer u voldoende bedenktijd heeft gehad (minimaal 1 dag na het ontvangen van deze folder), neemt de onderzoeker telefonisch contact met u op om te vragen of u wilt deelnemen aan dit onderzoek. Indien u heeft besloten deel te nemen, zullen wij u vragen om het bijgevoegde toestemmingsformulier te ondertekenen en mee te nemen naar de eerste afspraak bij de fysiotherapeut.

Wij willen u bedanken voor uw interesse in dit onderzoek, ook als u besluit om niet deel te nemen. Met vriendelijke groet,

Namens het hele onderzoeksteam,

D Strijker, arts‐onderzoeker, coördinerend onderzoeker e‐mail: [dieuwke.strijker@radboudumc.nl](mailto:dieuwke.strijker@radboudumc.nl)

# Bijlage A: contactgegevens

## Onderzoeksteam

D Strijker, arts‐onderzoeker, coördinerend onderzoeker Tel: (+31) (0)24 3610905

e‐mail: [dieuwke.strijker@radboudumc.nl](mailto:dieuwke.strijker@radboudumc.nl)

CJHM van Laarhoven, gastro‐intestinaal en oncologisch chirurg/afdelingshoofd Heelkunde, hoofdonderzoeker

Tel: (+31) (0)24 3616421

B van den Heuvel, gastro‐intestinaal en oncologisch chirurg, projectleider Tel: (+31) (0)24 3651560

Onafhankelijke expert

NP Riksen, internist/afdelingshoofd Interne Geneeskunde Tel: (+31) (0)24 3618819

Klachtenfunctionaris Radboud universitair medisch centrum

Instituut Waarborging Kwaliteit en Veiligheid Radboud universitair medisch centrum Huispostnummer 904 t.a.v. Klachtenfunctionaris

Antwoordnummer 540 6500 VC Nijmegen

Functionaris voor Gegevens‐bescherming

Radboud universitair medisch centrum

- - 1. Functionaris voor Gegevens‐bescherming Routenummer 624

Postbus 9101 6500 HB Nijmegen

e‐mail: [gegevensbescherming@radboudumc.nl](mailto:gegevensbescherming@radboudumc.nl)

Bijlage B: informatie over de verzekering

Voor de deelnemers aan dit onderzoek is door het Radboud universitair medisch centrum een verzekering afgesloten. Deze verzekering dekt schade door dood of letsel die het gevolg is van deelname aan het onderzoek, en die zich gedurende de deelname van de proefpersoon aan het onderzoek openbaart, of binnen vier jaar na beëindiging van diens deelname aan het onderzoek. De schade wordt geacht zich te hebben geopenbaard wanneer deze bij de verzekeraar is gemeld.

Bij schade kunt u direct contact leggen met de verzekeraar. De verzekeraar van het onderzoek is:

Onderlinge Waarborgmaatschappij Centramed B.A. Postbus 7374 2701 AJ Zoetermeer tel: 070‐3017070

e‐mail: schade@centramed.nl

De verzekering biedt een maximum dekking van € 650.000,‐ per proefpersoon en € 5.000.000,‐ voor het gehele onderzoek en € 7.500.000,‐ per jaar voor alle onderzoeken van dezelfde opdrachtgever.

Bovenstaande bedragen zijn opgenomen in het Besluit verplichte verzekering bij medisch‐ wetenschappelijk onderzoek met mensen. Informatie hierover kunt u vinden op de website van de Centrale Commissie Mensgebonden Onderzoek: [www.ccmo.nl.](http://www.ccmo.nl/)

De verzekering dekt schade die het gevolg is van het medisch‐wetenschappelijk onderzoek. De verzekering dekt niet:

- - - - Schade waarvan op grond van de aard van het onderzoek zeker of nagenoeg zeker was dat deze zich zou voordoen;
      - Schade aan de gezondheid die ook zou zijn ontstaan indien u niet aan het onderzoek had deelgenomen;
      - Schade door het niet (volledig) opvolgen van aanwijzingen of instructies door de proefpersoon;
      - Schade aan uw nakomeling(en), als gevolg van een nadelige inwerking van het onderzoek op u of uw nakomeling(en);
      - Schade door een bestaande behandelmethode bij onderzoek naar bestaande behandelmethoden;
      - Schade die een gevolg is van het optreden van een risico waarvoor u in de schriftelijke informatie bent gewaarschuwd, tenzij het risico zich in ernstiger mate voordoet dan was voorzien of het risico uiterst onwaarschijnlijk was.
