# Supplementary material for: Multimodal prehabilitation (Fit4Surgery) in high-impact surgery to enhance surgical outcomes: Study protocol of F4S PREHAB, a single center stepped wedge trial
Source: PLoS One. 2024 Jul 5;19(7):e0303829. doi: 10.1371/journal.pone.0303829 (PMC11226070; doi:10.1371/journal.pone.0303829)
Supplement: S1 Protocol — (PDF) [file pone.0303829.s002.pdf]

Multimodal intensive prehabilitation in high impact surgery

A stepped-wedge cluster randomized trial, from a patient's and hospital-efficiency perspective

Principal Investigator: CJHM van Laarhoven
Project leader: B van den Heuvel
Co-applicants: WJHJ Meijerink, MC Warlé

PROTOCOL TITLE: Multimodal intensive prehabilitation in high impact surgery – a stepped-wedge cluster randomized trial

**Short title**	F4S for high impact surgery
**EudraCT number**	Not applicable
**Version**	9
**Date**	15-11-2020
**Coordinating investigator**	D. Strijker (MD, MSc)

**Principal Investigator(s)**	<u>Department of Surgery, Radboudumc</u><br><br>CJHM van Laarhoven (MD, PhD, MSc (Ox))<br><br><u>Department of Operation Rooms, Radboudumc</u><br><br>B van den Heuvel (MD, PhD),<br>WJHJ Meijerink (MD, PhD)
	Research team Radboudumc Nijmegen:<br><br>1. Dr. Michiel C. Warlé<br>2. Prof. dr. Peter F.A. Mulders<br>3. Prof. dr. Ronald H.M.A. Bartels<br>4. Prof. dr. Marinus de Kleuver<br>5. Prof. dr. Wim J. Morshuis<br>6. Prof. dr. Stefaan J. Bergé<br>7. Prof. dr. Dietmar Ulrich<br>8. Prof. dr. Didi D.M. Braat<br>9. Prof. dr. Dietmar Ulrich<br>10. Prof. dr. Henri A.M. Marres<br>11. Dr. Larien M. Buffart<br>12. Dr. Manon G.A. van den Berg<br>13. Dr. Steven Teerenstra<br>14. Dr. Stefan J. van Rooijen<br>15. Prof. dr. Joost P.H. Drenth

**Sponsor**	Radboudumc, Nijmegen
**Subsidising party**	Radboudumc, Nijmegen
**Independent expert (s)**	Prof. dr. Niels P. Riksen
**Laboratory sites**	Department of Surgery research laboratory, Radboudumc
**Pharmacy**	Not applicable

PROTOCOL SIGNATURE SHEET

Name	Signature	Date
**Sponsor or legal representative:**<br>B Lahuis,<br>CEO board of directors Radboudumc		
**Head of Department of Surgery:**<br>CJHM van Laarhoven		10-11-2020
**Principal Investigator:**<br>CJHM van Laarhoven		10-11-2020

TABLE OF CONTENTS

1. INTRODUCTION AND RATIONALE 11
2. OBJECTIVES 15
3. STUDY DESIGN 16
4. STUDY POPULATION 18
   4.1 Population (base) 18
   4.2 Inclusion criteria 17
   4.3 Exclusion criteria 19
   4.4 Sample size calculation 21
5. TREATMENT OF SUBJECTS 23
   5.1 Investigational product/treatment 21
   5.2 Use of co-intervention 27
   5.3 Escape medication 27
6. INVESTIGATIONAL PRODUCT 28
7. NON-INVESTIGATIONAL PRODUCT 28
8. METHODS 29
   8.1 Study parameters/endpoints 29
      8.1.1 Main study parameter/endpoint 29
      8.1.2 Secondary study parameters/endpoints 29
      8.1.3 Other study parameters 30
   8.2 Randomization, blinding and treatment allocation 30
   8.3 Study procedures 31
   8.4 Withdrawal of individual subjects 34
      8.4.1 Specific criteria for withdrawal 34
   8.5 Replacement of individual subjects after withdrawal 34
   8.6 Follow-up of subjects withdrawn from treatment 34
   8.7 Premature termination of the study 34
9. SAFETY REPORTING 35
   9.1 Temporary halt for reasons of subject safety 35
   9.2 AEs, SAEs and SUSARs 35
      9.2.1 Adverse events (AEs) 35
      9.2.2 Serious adverse events (SAEs) 35
      9.2.3 Suspected unexpected serious adverse reactions (SUSARs) 36
   9.3 Annual safety report 36
   9.4 Follow-up of adverse events 36
   9.5 Data Safety Monitoring Board (DSMB) / Safety Committee 36
10. STATISTICAL ANALYSIS 38
   10.1 Primary study parameter(s) 38
   10.2 Secondary study parameter(s) 38
   10.3 Other study parameters 39
   10.4 Interim analysis 39
11. ETHICAL CONSIDERATIONS 40

11.1 Regulation statement 40
11.2 Recruitment and consent 40
11.3 Objection by minors or incapacitated subjects 40
11.4 Benefits and risks assessment, group relatedness 40
11.5 Compensation for injury 41
11.6 Incentives 41
12. ADMINISTRATIVE ASPECTS, MONITORING AND PUBLICATION 42
12.1 Handling and storage of data and documents 42
12.2 Monitoring and Quality Assurance 42
12.3 Amendments 42
12.4 Annual progress report 42
12.5 Temporary halt and (prematurely) end of study report 42
12.6 Public disclosure and publication policy 43
13. STRUCTURED RISK ANALYSIS 44
13.1 Potential issues of concern 44
13.2 Synthesis 44
14. APPENDICES 45
15. REFERENCES 46

LIST OF ABBREVIATIONS AND RELEVANT DEFINITIONS

1RM	One Repetition Maximum
ABR	ABR form, General Assessment and Registration form, is the application form that is required for submission to the accredited Ethics Committee; in Dutch: Algemene Beoordeling en Registratie
ACSM	American College of Sports Medicine
AE	Adverse Event
AL	Anastomotic Leakage
AR	Adverse Reaction
CA	Competent Authority
AT	Anaerobic Threshold
BIA	Bioelectrical Impedance Analysis
BMI	Body Mass Index
CCI	Comprehensive Complication Index
CCMO	Central Committee on Research Involving Human Subjects; in Dutch: Centrale Commissie Mensgebonden Onderzoek
CHAMPS	Community Healthy Activities Model Program for Seniors
CPET	Cardio Pulmonary Exercise Testing
CV	Curriculum Vitae
DAMP	Damage Associated Molecular Pattern
DSMB	Data Safety Monitoring Board
ECG	Electrocardiography
EDTA	Ethylenediamine Tetraacetic Acid
EORTC	European Organization for Research and Treatment for Cancer
ERAS	Enhanced Recovery After Surgery
EU	European Union
EudraCT	European drug regulatory affairs Clinical Trials
FFQ	Food Frequency Questionnaire
GCP	Good Clinical Practice
HADS	Hospital Anxiety and Depression Scale
HF	Heart Frequency
HIPEC	Hypothermia Intra Peritoneal Chemotherapy
HIIT	High Intensity Interval Training
HRQL	Health Related Quality of Life
IB	Investigator's Brochure
IC	Informed Consent
IL	Interleukin
IMP	Investigational Medicinal Product
IMPD	Investigational Medicinal Product Dossier
LOS	Length of Hospital Stay
LPS	Lipopolysaccharide
METC	Medical Research Ethics Committee (MREC); in Dutch: Medisch Ethische Toetsing Commissie
NRT	Nicotine Replacement Therapy
PG-SGA SF	Patient-Generated Subjective Global Assessment Short Form
PREHAB	Prehabilitation
QLQ	Quality of Life Questionnaire
RCT	Randomized Controlled Trial
REE	Resting Energy Expenditure
RQ	Respiratory Quotient
(S)AE	(Serious) Adverse Event
SPC	Summary of Product Characteristics; in Dutch: officiële productinfomatie IB1-tekst
Sponsor	The sponsor is the party that commissions the organization or performance of the research, for example a pharmaceutical company, academic hospital,

	scientific organization or investigator. A party that provides funding for a study but does not commission it is not regarded as the sponsor, but referred to as a subsidising party
SUSAR	Suspected Unexpected Serious Adverse Reaction
TNF	Tumor Necrosis Factor
Wbp	Personal Data Protection Act; in Dutch: Wet Bescherming Persoonsgevens
WMO	Medical Research Involving Human Subjects Act; in Dutch: Wet Medisch-wetenschappelijk Onderzoek met Mensen

SUMMARY

Rationale: High impact surgery has major consequences on the quality of life of patients. It takes them off normal work and life for prolonged periods and often they do not return to the preoperative level of daily activities and societal and work participation. Besides the regular impact of surgery, postoperative complications occur in up to 15-60% of patients and are associated with a higher mortality rate. The number and severity of complications is principally related to the initial quality of the surgical treatment. But also, it is strongly related to patient's individual pre-operative functional capacity, pre-operative physical fitness, nutritional status, mental health, immune status and intoxications like alcohol abuse and smoking.

Complex operations are the core business of academic surgical departments and the whole peri-operative process (surgery, anesthesiology, intensive care treatment), as post-operative morbidity and handling of complications is intensive and costly. Traditional approaches have mainly focused on minimizing operative trauma (minimal invasive operations) and peri-operative clinical recovery programs, such as the Enhanced Recovery After Surgery (ERAS) protocol. Recent evidence, however, shows that the preoperative period might be the optimal time frame for intervention to achieve short term and long lasting effects. During the last years, various so-called prehabilitation programs have been initiated with promising results.

Prehabilitation, the optimization of a patient preoperatively, seems to prevent postoperative complications, enhance recovery after surgery and reduce cost of the burden of care. Therefore prehabilitation promises to be a straightforward intervention with a clear positive intervention-outcome correlation from both patient's perspective and hospital's perspective. However, high levels of evidence lack, due to poor methodology and lack of a comprehensive approach of previous studies on the effect of prehabilitation. Moreover, the mechanistical effects of prehabilitation have not been explained so far and the effects on a macro-economic level are not clear.

Objective/study design: A stepped-wedge cluster randomized trial with a clear aim to demonstrate the effects on clinical outcomes, the underlying mechanistical effect and the cost efficiency of prehabilitation across a wide range of patients, diseases and procedures.

Study population: Patients undergoing elective high impact surgery for colon cancer, rectal cancer, esophageal cancer, liver metastases from colorectal cancer, pancreato-biliary cancer, peritoneal carcinomatosis from colorectal cancer, abdominal aortic aneurysm, renal cancer, bladder cancer, supratentorial meningioma, hip arthrosis, osteosarcoma, pulmonary cancer, thoracic aortic aneurysm, head and neck cancer, mouth cancer, breast reconstruction, ovarian cancer or endometriosis will be included in this study.

Intervention: Patients will undergo a multimodal intensive prehabilitation program prior to high impact surgery including an exercise program, a nutritional intervention, psychological support and smoking cessation support.

Main study parameters/ endpoints:

This study investigates the effect of prehabilitation on:

- Individual patient's level: postoperative complications, length of hospital stay, physical fitness, nutritional status, mental health, intervention adherence
- Mechanistic level: immune status
- Hospital-efficiency level: costs due to complications, costs due to length of hospital stay, budget impact analysis, cost-effectiveness
- Macro-economic level: changes in patients volumes, shifts in care between 2nd and 1st line healthcare

1. INTRODUCTION AND RATIONALE

Surgical treatment is the basis in multimodal treatment regimens for most of oncological diagnoses. Also in other fields like cardiovascular disease, surgical treatment or endovascular treatment forms the basis and have an high impact on quality of life. Despite a high quality of the interventions and developments like minimal invasive laparoscopic and robotic techniques, postoperative complications occur in 15-60% of patients and are associated with a higher mortality rate, a lower Health Related Quality of Life (HRQoL), prolonged hospital stays and increased hospital costs(1-3). Even in the absence of complications, major surgery is associated with a 20-40% reduction in functional capacity(4). Compromised functional capacity is associated with preoperative health status, preoperative fatigue, weight, grip strength, the degree of surgical trauma and intensity of metabolic response.

Traditional approaches, such as minimal invasive surgery (laparoscopy) and the ERAS program(5), targeted the peri-operative and post-operative period to improve outcomes in patients undergoing surgery. However, the (early) post-operative period may not be the best time for patients to make significant changes in their dietary intake and functional mobility, as patients are physically unfit and distressed about the healing process and possible additional treatments for their underlying condition.

The pre-operative period therefore may in fact be a better time to intervene in the patient-related factors that contribute to physical recovery and alleviation of emotional distress for the surgery and recovery process(6). This process of enhancing functional capacity of the individual to enable him or her to withstand an incoming stressor has been termed *prehabilitation(7, 8)*.

Prehabilitation is a specific form of lifestyle intervention. Lifestyle interventions, as a part of the broader prevention program, are considered an important tool in increasing population's health and decreasing health care consumption. Whereas primary and secondary prevention find difficulty to achieve sustainable effects, prehabilitation -as tertiary prevention- explicitly has the potential to render a measurable effect on three levels. Healthy people often find it difficult to catch up with life style changes in primary and secondary prevention programs for long sustainable periods. Patients facing treatment for actual diseases, on the other hand, are maximal motivated and eagerly adopt to prehabilitation programs. It both reinforces patients in 'being in control' of the process, and it provides them a so-called 'teachable moment' to improve health behavior(9).

Besides improved recovery outcomes after surgery, patients may also benefit from prehabilitation during chemo- and radiotherapy. If a patient's functional capacity is improved preoperatively, postoperative recovery might be faster and adjuvant chemotherapy might be started earlier. Also, completion rates of chemotherapy might increase. This is important since previous studies showed that early start and completion of chemotherapy resulted in an increase in survival(10). A recent small randomized clinical trial showed that personalized prehabilitation in major

abdominal surgery reduced complications by 51% and the rate of complications per patient from 1.4 to 0.5(11). While some level of prehabilitation has already been introduced informally in many Dutch surgical departments since 2015, robust programs have not been implemented and a clear understanding of how prehabilitation achieves its effect on clinical outcomes is lacking. Knowledge of these underlying mechanisms is essential to further improve prehabilitation programs. On top of this, the positive clinical outcomes in terms of complication reduction and shortening of stay might possibly be overrated, while the methodological quality of studies is weak. Therefore, this comprehensive study will be focusing on different aspects than merely clinical outcomes to understand prehabilitation mechanistically and to evaluate the true benefit of an intensive and personalized prehabilitation program.

It is well known that several preoperative factors are strongly associated with complications after high impact surgery. The most important ones are: functional status, physical fitness, nutritional status, immune status, cigarette smoking and mental health. These are discussed below in detail, since the proposed prehabilitation program contains interventions to mitigate the impact of these factors on postoperative outcomes.

Physical fitness

It has been shown that poor baseline physical fitness increases the risk of complications after major non cardiac surgery and prolongs recovery after surgery. A review indicated that optimizing functional exercise capacity in the surgical population can result in fewer postoperative complications, shortening of the length of hospital stay, reduction of disability, and improvement of quality of life(12). Based on the notion that preoperative exercise would have an impact on recovery of functional capacity after surgery, a randomized controlled trial has been performed by Carli et al(8). Results showed that patients whose functional capacity improved preoperatively, recovered well in the postoperative period. These results support that exercise prehabilitation is an effective way to improve recovery after surgery.

Nutritional status

The role of nutritional status in surgical recovery cannot be underestimated. Malnutrition with unintended weight loss and/or low lean muscle mass (sarcopenia) in surgery patients is associated with more post-operative complications, delayed recovery of bowel function, prolonged length of hospital stay, higher re-admission rates, higher costs, reduced quality of life, and higher incidence of postoperative morbidity and mortality(13-15). Furthermore, patients with cancer, the presence of low lean body mass may be related to lower tolerance to (neo)adjuvant therapy(16, 17).

Sarcopenia and sarcopenic obesity (sarcopenia in combination with high body weight) are associated with reduced muscle strength, functional decline, physical disability, increased risks of fractures and falls, enhanced risk of metabolic disease, increased length of hospital

stay and decreased survival in general populations of older adults(16, 18-20).
Preoperative nutritional intervention in malnourished patients with unplanned weight loss to improve a patient's nutritional status, has been shown an effective strategy for reducing risk of post-surgery complications such as infections and anastomotic leaks(13).

Cigarette smoking

Cigarette smoking itself is a well-known risk factor for postoperative complications. Smoking has a transient effect on the tissue microenvironment and a prolonged effect on inflammatory and reparative cell functions leading to delayed healing and complications(21). Wound contraction and collagen metabolism are also affected by a smoking-induced alteration in vitamin C turnover and change in inflammatory cell response(22). Evidence exists that preoperative smoking interventions reduce postoperative morbidity. Moreover, smoking cessation restores the tissue microenvironment rapidly and the inflammatory cellular functions within 4 weeks. Recent results show that smoking cessation 4 to 8 weeks prior to surgery significantly reduces postoperative complications(23).

Mental health

Psychological status may also play an important role in surgical recovery. It is well documented that patients awaiting major surgery experience anxiety concerning their upcoming operation, its outcome, and their course of healing and recovery(24, 25). They may also feel depressed, hold unrealistic expectations about their health status, and possess inadequate strategies for coping with pre- and post- operative periods. Any of these factors may influence pain(24) and interfere with post-operative functioning(26, 27). Therefore, psychological interventions prior to surgery might have a positive effect on postoperative recovery.

Multimodal intensive personalized prehabilitation

Although exercise, nutritional interventions, smoke cessation and psychological interventions prior to surgery have separately shown to improve several post-surgical outcome parameters, only few studies have been performed investigating a combined multimodal intervention approach in preoperative complex surgical patients. Since it has been established that the number and severity of complications is closely related to preoperative physical fitness, nutritional status, smoking behavior and psychological well-being, there is a growing need to target these issues by means of the implementation of a multimodal intervention program. It is well known that there is a strong interaction between exercise and nutrition. Their synergistic effects on muscle protein synthesis have been associated with gains in muscle mass, increased muscle strength, improved functional capacity and better functional performance(19).From a physiological point of view and based on limited practical experience, it seems feasible to achieve clinically relevant effects during the frame of 4-6 weeks between diagnosis and operation. However, this will only

be achieved if targeted interventions involving exercise, nutrition, smoke cessation and psychological support are implemented(28-32).

Immune status

Postoperative infections represent a major part of all complications within the first period after surgery(33-35). During and after major surgery hyperinflammatory responses, initiated by cytokine production, are accompanied and/or followed by counterregulatory anti-inflammatory responses. This can cause a dysfunctional state in which immune cells are unable to adequately respond to pathogens. This phenomenon, called surgery- or trauma- induced immunoparalysis, is associated with increased susceptibility to infectious complications after surgery, along with an increased mortality rate(36, 37).

As showed in the figure below, it is hypothesized that improving physical fitness by prehabilitation 1) improves the preoperative immune status and therefore 2) reduces the degree of immunoparalysis during and after major surgery. This might lead to less infectious complications after high impact surgery. Measuring the degree of immunoparalysis prior to prehabilitation, during and after high impact surgical procedures might give insight into the mechanistical effect of prehabilitation.

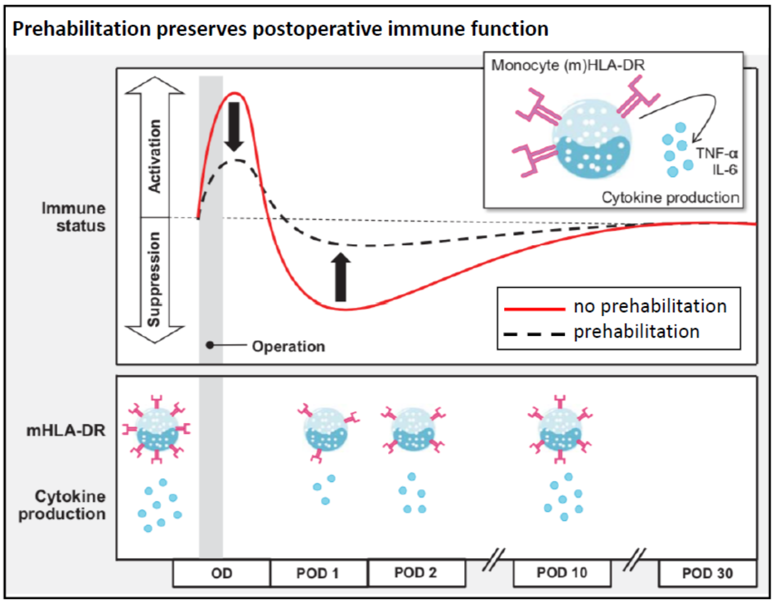

2. OBJECTIVES

Primary Objective: To determine the effects of a multimodal intensive prehabilitation program a high-risk patient group on complications (Clavien Dindo score and Comprehensive Complication Index (CCI) score)

Secondary Objective(s): To investigate the effects of multimodal intensive personalized prehabilitation on an:

- Individual patient's level, i.e. length of hospital stay (in days), physical fitness (estimated VO_2 peak, indirect 1 repetition measures, physical activity), nutritional status (body weight, fat-free mass, Patient-Generated Subjective Global Assessment (PG-SGA), mental health (HRQoL), intervention adherence
- Mechanistic level, i.e. innate immune response (degree of immunoparalysis)
- Hospital-efficiency level: costs due to complications, costs due to length of hospital stay, budget impact, cost-effectiveness
- Macro-economic level: changes in patients volumes, shifts in care between 2nd and 1st line healthcare

3. STUDY DESIGN

This is a monocenter stepped-wedge cluster randomized trial of patients aged >16 years undergoing elective high impact surgery in Radboudumc, Nijmegen. Patients will be grouped into 20 clusters based on diagnosis; colon cancer, rectal cancer, esophageal cancer, liver metastases from colorectal cancer, pancreato-biliary cancer, peritoneal carcinomatosis from colorectal cancer, abdominal aortic aneurysm (open and endovascular), renal cancer. bladder cancer, supratentorial meningioma, hip arthrosis, osteosarcoma, pulmonary cancer, thoracic aortic aneurysm, head and neck cancer, mouth cancer, breast reconstruction, ovarian cancer or endometriosis. The prehabilitation program intervention will commence in one cluster each month with the order of clusters determined by computer based randomization. The stepped-wedge design allow delivery of the intervention at an organizational level with evaluation of outcome measures at a patients level. Structuring the prehabilitation program intervention through a staged activation in a random order provides important methodological advantages. The design allows to control adoption bias and to adjust for time-based changes in the background level of patient care in statistical analysis. A key strength of stepped-wedge design is that the prehabilitation program can be offered to every patient who is undergoing elective high impact surgery in Radboudumc, Nijmegen.

This stepped-wedge cluster randomized trial will be combined with an observational cohort study performed in Radboudumc on surgery induced danger-associated molecule patterns (DAMPs) and innate immune changes in patients undergoing endovascular aneurysm repair(38). Patients within the care path ‘abdominal aortic aneurysm’ will undergo additional blood tests, according to protocol, to determine the effect of prehabilitation on the innate immune response (see also 8. METHODS).

Cluster	Pre (24 months)				Switching (20 months)																				Post (4 months)			
1	0	…	…	0	1	1	1	1	1	1	1	1	1	1	1	1	1	1	1	1	1	1	1	1	1	1	1	1
2	0	…	…	0	0	1	1	1	1	1	1	1	1	1	1	1	1	1	1	1	1	1	1	1	1	1	1	1
3	0	…	…	0	0	0	1	1	1	1	1	1	1	1	1	1	1	1	1	1	1	1	1	1	1	1	1	1
4	0	…	…	0	0	0	0	1	1	1	1	1	1	1	1	1	1	1	1	1	1	1	1	1	1	1	1	1
5	0	…	…	0	0	0	0	0	1	1	1	1	1	1	1	1	1	1	1	1	1	1	1	1	1	1	1	1
6	0	…	…	0	0	0	0	0	0	1	1	1	1	1	1	1	1	1	1	1	1	1	1	1	1	1	1	1
7	0	…	…	0	0	0	0	0	0	0	1	1	1	1	1	1	1	1	1	1	1	1	1	1	1	1	1	1
8	0	…	…	0	0	0	0	0	0	0	0	1	1	1	1	1	1	1	1	1	1	1	1	1	1	1	1	1
9	0	…	…	0	0	0	0	0	0	0	0	0	1	1	1	1	1	1	1	1	1	1	1	1	1	1	1	1
10	0	…	…	0	0	0	0	0	0	0	0	0	0	1	1	1	1	1	1	1	1	1	1	1	1	1	1	1
11	0	…	…	0	0	0	0	0	0	0	0	0	0	0	1	1	1	1	1	1	1	1	1	1	1	1	1	1
12	0	…	…	0	0	0	0	0	0	0	0	0	0	0	0	1	1	1	1	1	1	1	1	1	1	1	1	1
13	0	…	…	0	0	0	0	0	0	0	0	0	0	0	0	0	1	1	1	1	1	1	1	1	1	1	1	1
14	0	…	…	0	0	0	0	0	0	0	0	0	0	0	0	0	0	1	1	1	1	1	1	1	1	1	1	1
15	0	…	…	0	0	0	0	0	0	0	0	0	0	0	0	0	0	0	1	1	1	1	1	1	1	1	1	1
16	0	…	…	0	0	0	0	0	0	0	0	0	0	0	0	0	0	0	0	1	1	1	1	1	1	1	1	1
17	0	…	…	0	0	0	0	0	0	0	0	0	0	0	0	0	0	0	0	0	1	1	1	1	1	1	1	1
18	0	…	…	0	0	0	0	0	0	0	0	0	0	0	0	0	0	0	0	0	0	1	1	1	1	1	1	1
19	0	…	…	0	0	0	0	0	0	0	0	0	0	0	0	0	0	0	0	0	0	0	1	1	1	1	1	1
20	0	…	…	0	0	0	0	0	0	0	0	0	0	0	0	0	0	0	0	0	0	0	0	1	1	1	1	1

Retrospectively, 24 months of pre-measurements will be collected. The prospective data collection will include 20 months in which every month one cluster switches and 4 months of post-measurements. In the above a '0' indicates the control condition and a '1' the intervention condition.

4. STUDY POPULATION

4.1 Population (base)

Patients undergoing 20 types of elective high impact surgical procedures, as shown in the table below, in Radboudumc will be included in this study.

Table 1. Type of surgical procedure per diagnosis

Diagnosis	Surgical procedure
Colon cancer	Colon resection
Rectal cancer	Rectal resection
Esophageal cancer	Esophageal resection
Liver metastases from colorectal cancer	Liver resection
Pancreato-biliary cancer	Pancreas resection
Peritoneal carcinomatosis from colorectal cancer	HIPEC
Abdominal aortic aneurysm (open)	Open abdominal aortic aneurysm repair
Abdominal aortic aneurysm (endovascular)	EVAR
Renal cancer	Nephrectomy
Bladder cancer	Cystectomy
Supratentorial meningioma	Craniotomy
Hip arthrosis	Total hip arthroplasty
Osteosarcoma	Limb salvage procedure
Pulmonary cancer	Lung resection
Thoracic aortic aneurysm	Open thoracic aortic aneurysm repair
Head and neck cancer	Laryngectomy
Mouth cancer	Mandibular resection or reconstruction
Breast reconstruction	DIEP flap reconstruction
Ovarian cancer	Hysterectomy
Endometriosis	Excision of endometriosis

4.2 Inclusion criteria

In order to be eligible to participate in this study, a subject must meet all of the following criteria:

- o Patients >16 years,
- o scheduled for elective high impact surgery,
- o independent of (neo-) chemotherapy and/or radiotherapy,
- o obtained written informed consent.

4.3 Exclusion criteria

A potential subject who meets any of the following criteria will be excluded from participation in this study:

- Metastatic disease known preoperatively (exemption colorectal carcinoma liver metastases and peritoneal carcinomatosis from colorectal or ovarian cancer),
- paralytic or immobilized patients who are not able to complete exercise intervention,
- premorbid conditions or orthopedic impairments which contraindicate exercise,
- cognitive disabilities,
- unstable cardiac or respiratory disease,
- renal failure stage 3 or higher,
- ASA score 4 or higher,
- illiteracy (disability to read and understand Dutch).

A patient accrual rate of 60-80 percent is estimated.

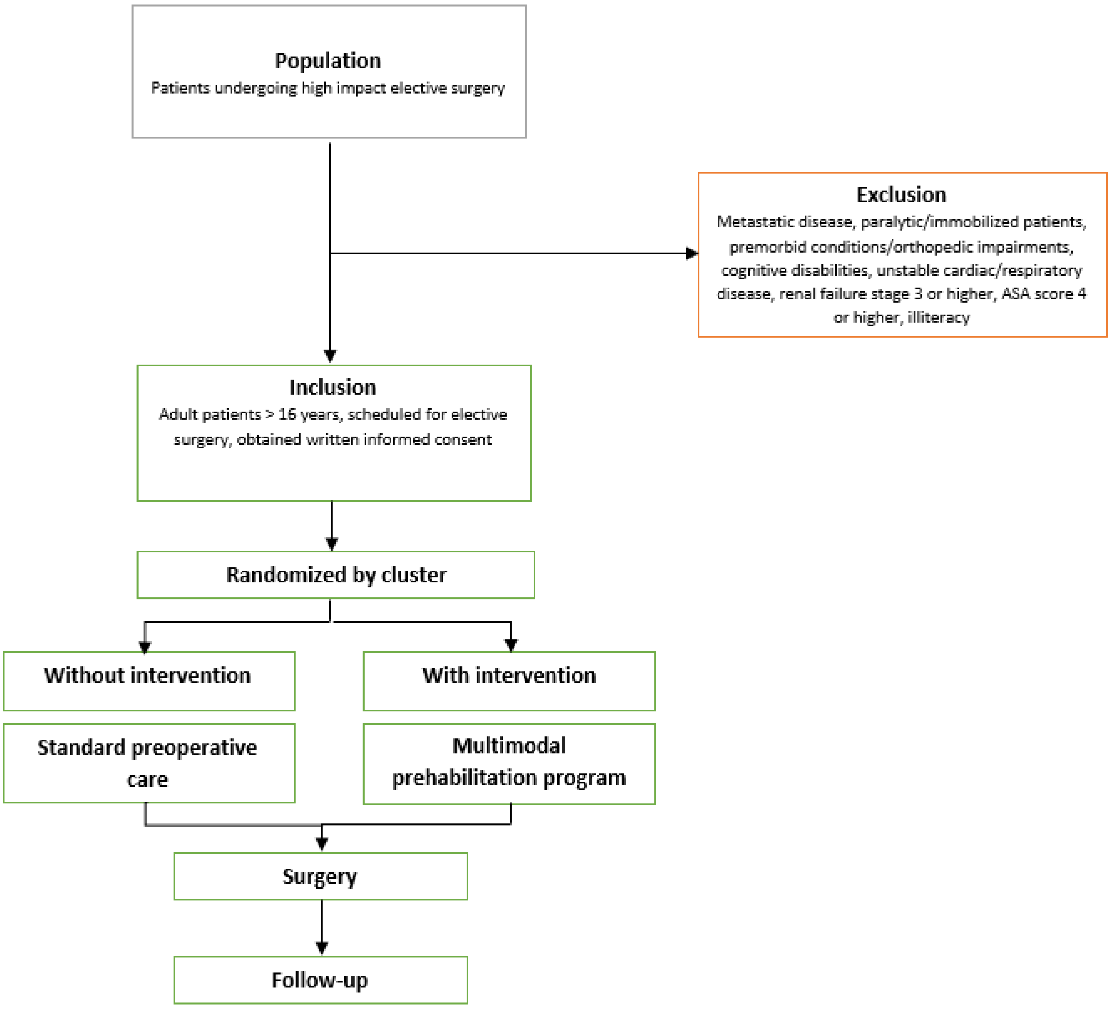
Population
Patients undergoing high impact elective surgery
Exclusion
Metastatic disease, paralytic/immobilized patients, premorbid conditions/orthopedic impairments, cognitive disabilities, unstable cardiac/respiratory disease, renal failure stage 3 or higher, ASA score 4 or higher, illiteracy
Inclusion
Adult patients > 16 years, scheduled for elective surgery, obtained written informed consent
Randomized by cluster
Without intervention
With intervention
Standard preoperative care
Multimodal prehabilitation program
Surgery
Follow-up

4.4 Sample size calculation

We consulted Dr. Steven Teerenstra, department of Epidemiology, Radboudumc for the sample size calculation.

The calculation for this stepped-wedge cluster randomized trial is based on 20% relative reduction in the percentage of Clavien-Dindo score II or higher.

Table 2. Numbers of surgical procedures/year and overall complication rates in Radboudumc

Surgical procedure	Numbers of surgical procedures/year	Overall complication rates (Clavien-Dindo II or higher)
Colon resection	65	15%
Rectal resection	60	15%
Esophageal resection	80	55%
Liver resection	100	15%
Pancreas resection	78	33%
HIPEC	40	30%
Open abdominal aortic aneurysm repair	35	58%
EVAR	120	24%
Nephrectomy	60	25%
Cystectomy	78	38%
Craniotomy	55	11%
Total hip arthroplasty	50	10% (based on literature)
Limb salvage procedure	30	30% (based on literature)
Lung resection	60	20% (based on literature)
Open thoracic aortic aneurysm repair	150	10% (based on literature)
Laryngectomy	65	30% (based on literature)
Mandibular resection or reconstruction	60	35% (based in literature)
DIEP flap reconstruction	118	22%
Hysterectomy	50	10% (based on literature)
Excision of endometriosis	50	30% (based on literature)

Sample size was based on simulations in a log-binomial model with cluster (surgical procedure), intervention, and time (as continuous variable) as fixed effects. Monthly binary data were generated per cluster (surgical procedure) per month with a binary distribution according to the sample size corresponding to Table 2 with a small overall time trend. The percentage complications in the control condition was based on Table 2, and in the intervention condition this percentage had a relative reduction of 20% (e.g., nephrectomy was assumed to change from 25% to 20%). Based on the simulations, power was 83%.

Based on actual recruitment, the number of retrospectively pre-measurements and prospectively collected post-measurements may be increased (see 10.4). The total prospective recruitment in terms of patients is expected to be 2828 (2 year times 1414 patients/year), although the power will not primarily be driven by the total number of patients but rather their distribution over clusters over time.

5. TREATMENT OF SUBJECTS

5.1 Investigational product/ treatment

1. Inclusion

Information on the inclusion procedure is given in section 11.2 Recruitment and consent.

2. Preoperative period

A. Without intervention (standard preoperative care)

Patients will undergo standard preoperative care according to Dutch guidelines(39). To collect data, two additional appointments will be combined with regular, preoperative outpatient clinic visits. Patients will undergo measurements regarding physical fitness, nutritional status and health status as shown in Appendix I.

B. With intervention (multimodal intensive prehabilitation program)

Additionally to the standard preoperative care, patients will undergo a multimodal intensive prehabilitation program prior to the scheduled elective procedure, including an exercise program, nutritional intervention, smoking cessation and psychological support. Patients will undergo prehabilitation throughout the full preoperative period. The surgical procedure will not be delayed due to this program. Since the preoperative period differs between the clusters regarding time and treatment, the time period for prehabilitation is not the same within all clusters. Therefore, patients will be divided into two groups based on their diagnosis and preoperative treatment as shown in the table below.

Table 3. Groups based on diagnosis and preoperative treatment

**Group: PREHAB LONG**<br>**Duration prehabilitation: >5 weeks**	**Group: PREHAB SHORT**<br>**Duration prehabilitation: <5 weeks**
Pancreato-biliary cancer (after completing neo-adjuvant therapy)	Hepatic colorectal cancer metastases
Rectal cancer (after completing neo-adjuvant therapy)	Peritoneal carcinomatosis from colorectal cancer
Esophageal cancer (after completing neo-adjuvant therapy)	Colon cancer
Supratentorial meningioma	Abdominal aortic aneurysm (open and endovascular)
Hip arthrosis	Renal cancer
Osteosarcoma	Bladder cancer
Pulmonary cancer (after completing neo-adjuvant therapy)	Pancreato-biliary cancer without neo-adjuvant therapy
	Pulmonary cancer without neo-adjuvant therapy
	Thoracic aortic aneurysm

	Head and neck cancer
	Breast reconstruction
	Mouth cancer
	Ovarian cancer
	Endometriosis

Despite differences in prehabilitation duration, general goals apply for each participating patients. The different disciplines (exercise program, nutritional intervention, psychological support, smoking cessation) involved in the prehabilitation program are described below.

2.B.1 Triage

Triage will be performed focusing on anemia and disturbed glucose regulation. In case of abnormal blood levels patients will be referred to an internist. Patients will also be screened on frailty using the Geriatric 8 questionnaire. A geriatrist will be consulted in case a patient is screened at risk. The prehabilitation program will start after approval of the consulted physician.

2.B.2 Exercise program

- Screening: all patients undergoing the multimodal intensive prehabilitation program will be screened for physical fitness according to the American College of Sports Medicine (ACSM) exercise preparticipation health screening questionnaire for exercise professionals. Patients screened at risk will be referred to a cardiologist or pulmonologist to undergo additional tests (e.g. ECG, spirometry, cardiopulmonary exercise test) to assess whether undergoing high intensity training (HIIT) during prehabilitation is safe.
- Assessment: the content of the exercise program will be adjusted based on a patient's estimated VO_2 peak (submaximal Astrand-Rhyming test) and muscle strength (indirect 1RM).
- Goal: to increase physical fitness (estimated VO_2 peak and indirect 1RM) by 10%.
- The content of the exercise program: to reach the goal, the exercise program consists of the following components:
    - *Endurance training:*
        - High intensity interval training (HIIT)
            - This supervised training (by 1st line physiotherapist) will be done
                - Group 'PREHAB LONG': two times a week
                - Group 'PREHAB SHORT': three times a week
            - The interval training duration is 28 minutes and performed with 4 intervals of moderate intensity (3 minutes) and 4 intervals of high intensity (4 minutes)
            - The workload is dosed at a percentage of estimated VO_2 peak. High intensity is considered 90% of estimated VO_2 peak
            - This interval training aims to reach levels of Borg 15-17 and >85%

of the age-predicted maximum heart rate, as long as the patient could effort the exercise

- Exercise may be performed on a bicycle, a rower, a treadmill and/or on other aerobic exercise machines

o *Resistance training*

- This supervised training (by 1st line physiotherapist) will be done
    - Group ‘PREHAB LONG’: two times a week
    - Group ‘PREHAB SHORT’: three times a week
- Strength training will be the same concept for all patients irrespective of VO_2 peak
- The strength exercises are performed according to: 2 seconds of concentric strength and 2 seconds of eccentric strength.
- The strength training consists of two series of 10 repetitions of six exercises: leg press, chest press, abdominal crunch, lat pull down, low row and step up
    - In week 1 using 65% of calculated indirect 1RM (measured during assessment)
    - In week 2 using 70% of calculated indirect 1RM (measured during assessment)
    - In week 3 using 75% of calculated indirect 1RM (measured during assessment)
    - In week 4 using 80% of calculated indirect 1RM (measured during assessment)
- The last bout with 10 repetitions needs to be attainable. If this is not the case in the next session dosing will be 5-10% lower. If in the last bout it appears that exercises are to low (≥15 repetitions will be achieved), in the next sessions dosing will be 5-10% higher

o *Homework/counseling*

- The homework will be done at the days without supervised training
- Patients are instructed to aim at 60 minutes walking and/or cycling a dayIf possible, patients can do more than 60 minutes of walking or cycling every day
- In case of lower exercise capacity it is advised to walk/cycle 2-3 times a day for periods of 20 minutes
- Also an electric bicycle, a stationary bicycle and/or a walking aid

(walker) may be necessary

- Instructions on breathing techniques will be given to reduce the risk of developing pneumonia postoperatively

- o *Rest*
  - Patients are instructed to take care of recovery and adequate rest and sleep
  - From 3 days before surgery no high intensity interval training and strength training are done. The patient will continue with the homework/counseling (walking/cycling every day) and the dietary supplements

2.B.3 Nutritional intervention

- Screening: a registered dietician will screen for a patient's nutritional status by Patient-Generated Subjective Global Assessment (PG-SGA).
- Assessment: a patient's body weight, length, 3-day food diary, hand grip strength and fat-free mass measured by Bioelectrical Impedance Analysis (BIA) are used to adjust the content of the nutritional intervention.
- Goal: to enhance the anabolic effect of physical training, to improve the lean body mass and to obtain or maintain an optimal nutritional status preoperatively
- The content of nutritional intervention includes:
  - o *Dietary advice*
    - ▪ Aiming for optimal energy intake bases on screening and assessment
    - ▪ Aiming for a protein intake of 1.5 – 1.8 g/kg daily
  - o *Protein supplementation*
    - ▪ 30 g protein supplementation immediately following supervised training (within 1 hour) and before going to sleep
  - o *Vitamin supplementation*
    - ▪ Vitamin D:
      - Female patients, age <50 years with colored skin and/or little sun exposure
        - o 10 µg vitamin D daily
      - Female patients, age 50-69 years
        - o 10 µg vitamin D daily
      - Male patients, age <70 years
        - o 10 µg vitamin D daily
      - Female/male patients, age >70 years
        - o 20 µg vitamin D daily
    - ▪ Multivitamin: 50% of the recommended daily allowance supplementation

2.B.4 Psychological support

- Screening/assessment: psychological factors that may affect postoperative outcomes adversely will be identified by the Hospital Anxiety and Depression Scale (HADS).

- The content of psychological support: patients screened at risk will be seen by a trained psychologist to address a patient's anxieties, coping strategies and post-operative expectations. The goal of psychological support is to optimize psychological well-being and to provide ways of coping with surgery. If indicated, more session will follow preoperatively.

2.B.4 Smoking cessation

A smoke cessation program including intensive counseling and nicotine replacement therapy (NRT) will be offered to all patients during the weeks prior to the surgery. Approximately 15-20% of the patients in the study group are current smokers at the moment of cancer diagnosis. The goal is to achieve a smoke cessation percentage of 80.

3. Surgery

Surgery will be performed in the various departments of the Radboudumc by specialized attending surgeons. Surgical approach, including laparoscopic, endoscopic or open surgery will be at the discretion of the surgeon. Perioperative care will be followed by the Enhanced Recovery After Surgery (ERAS) program, which is an evidence-based multidisciplinary care plan(40). The ERAS program has been standardized care since more than 10 years now, and includes patient education, preoperative feeding, selected bowel preparation, multimodal analgesia, maintenance of perioperative normothermia, early oral intake and early mobilization, early removal of catheters and drains, and a pre-planned hospital stays according to clinical pathways.

4. Post-operative period

After discharging, patients will receive standard postoperative care according to Dutch guidelines(41). Questionnaires will be additionally performed at 6 and 12 months after surgery.

A 'subway line' map for each cluster will be designed to give a clear overview of treatment including the prehabilitation program for patients and health care workers.

5.2 Use of co-intervention

Not applicable

5.3 Escape medication

Not applicable

6. INVESTIGATIONAL PRODUCT

Not applicable

7. NON-INVESTIGATIONAL PRODUCT

Not applicable

8. METHODS

8.1 Study parameters/endpoints

8.1.1 Main study parameter/endpoints

Primary outcomes that are studied in this trial include:

Outcome	Tool used	Expressed in	Measured at
Postoperative complications	Clavien-Dindo classification	Score I-V	Surgery +4 weeks
	Comprehensive Complication Index	Score	Surgery +4 weeks

Table 4. Primary study parameters

8.1.2 Secondary study parameters/endpoints

The following secondary outcomes are studied in this cohort study:

Outcome	Tool used	Expressed in	Measured at
Length of hospital stay (LOS)	-	In days	Surgery +4 weeks
Change in physical fitness and activity	Estimated VO_2 peak (submaximal Astrand-Rhyming test)	Milliliters/kilogram/minute	Baseline<br>Surgery -1 week
	Indirect 1 repetition measures (1RM)	Weight in kilograms	Baseline<br>Surgery -1 week
	Physical activity (Short Questionnaire to Assess Health-enhancing physical activity (SQUASH)	Minutes per week	Baseline<br>Surgery +6 months<br>Surgery +12 months
Change in nutritional status	Body weight	In kilograms	Baseline<br>Surgery -1 week
	Fat-free mass (Bio Impedance Analysis (BIA)	Weight in kilograms	Baseline<br>Surgery -1 week
	PG-SGA	Score (A, B or C)	Baseline<br>Surgery -1 week

Change in mental health	Health-related Quality of Life (SF-36)	Score	Baseline<br>Surgery -1 week<br>Surgery +6 months<br>Surgery +12 months
	Health-related Quality of Life (EQ-5D-5L)	Score	Surgery +6 months
Change in innate immune response *(only in abdominal aortic aneurysm group)*	Degree of immunoparalysis	Monocyte HLA-DR (mHLA-DR) expression	Baseline<br>Prior to anesthesia<br>Surgery +2 days<br>Surgery +6 weeks
Preoperative health behavior	Questionnaire for patients in control group<br>Questionnaire for patients in intervention group		Surgery -1 week
Intervention adherence			Surgery -1 week
Costs due to complications			Surgery +4 weeks
Costs due to length of hospital stay			Surgery +4 weeks
Budget impact analysis			Surgery +1 year
Cost-effectiveness			Surgery +1 year
Changes in patients volumes			Surgery +1 year
Shifts between 2nd and 1st line healthcare			Surgery +1 year

Table 5. Secondary study parameters

A more detailed description of the outcome parameters is given in section 8.3 Study procedures.

8.1.3 Other study parameters

Not applicable

8.2 Assignment to study

Patients eligible for surgery, according to inclusion- and exclusion criteria, will be assigned to either standard preoperative care or the multimodal intensive prehabilitation program, depending on the time frame and cluster in which the patient is included.

8.3

8.4 Study procedures

Study procedures are performed in the Radboudumc departments of Surgery, Urology, Neurosurgery, Orthopaedics, Head and neck surgery, Plastic surgery, Cardiothoracic surgery, and Gynaecology, as well as in the research lab of the department of surgery. Life style assessment and questionnaires will be taken before surgery and post operatively up to one year. An overview of all measurements performed during this stepped-wedge cluster randomized trial are demonstrated in Appendix I.

- The Clavien-Dindo classification is correlated with the complexity of the operation as well as the length of hospitalization and may be used internationally as quality screening for surgery(42, 43). The Comprehensive Complication Index summarizes the postoperative wellbeing of the patient concerning complications based on the Clavien-Dindo classification(44). The CCI-score is easily assessed at www.assessurgery.com. Both scores will be determined at four weeks after surgery.

- Screening for physical fitness will be done according to the American College of Sports Medicine's (ACSM) guidelines for Exercise Preparticipation Health Screening (45) to assess whether high intensity interval (HIT) training during prehabilitation is safe. The screening process is based on current levels of structured physical activity, the presence of major signs or symptoms suggestive of cardiovascular, pulmonary or metabolic diseases, and de desired exercise intensity. These guidelines require the exercise professional to 1) complete a cardiovascular risk factor profile; 2) determine if the patient has known cardiovascular, pulmonary, and/or metabolic diseases; and 3) identify major signs or symptoms suggestive of cardiovascular, pulmonary, and/or metabolic diseases. With this information a patient is classified as low, moderate, or high risk. Patients screened at high risk will be referred to a cardiologist or pulmonologist to get medical clearance for HIT training during prehabilitation. The exercise preparticipation health screening questionnaire for exercise professionals(46), developed to simplify the ACSM guidelines, will be performed prior to start of the prehabilitation program.

- The submaximal Astrand-Rhyming test will be used to estimate a patient's cardiorespiratory fitness. It is a simple, 6-minute, non-maximal cycle ergometer test(47) and therefore safer and better tolerated than maximal testing procedures, irrespective of the accuracy of the VO2 peak prediction(48).

- The indirect 1 repetition measures (1RM) are used to dose the training and will be determined based on chest press and leg press to assess the impact of the intervention throughout the perioperative period(49). The 1RM is calculated by the Brzycki formula: 1RM=W*36/(37-r) (w=weight, r=repetitions)(50).

- The Patient-Generated Subjective Global Assessment (PG-SGA) screening tool will be used to identify malnutrition. This is an internationally validated instrument and preferred screening method to identify malnutrition in oncologic patients(20, 51-54). The energy requirements of the patient. Is calculated by the WHO formula.. Håkonsen et al recommended the PG-SGA as a nutritional assessment tool in colorectal cancer patients in combination with other parameters(55). The PG-SGA consists of two parts: 1) the first part is the Patient Generated Subjective Global Assessment Short Form (PG-SGA SF) which includes a brief questionnaire addressing current weight, weight history, acute weight changes, changes in food intake over the past month, occurrence of nutrition impact symptoms experienced over the previous 2 weeks and changes in physical activities and functions over the previous month. The PG-SGA SF will be completed by patients themselves and generates a score for nutrition risk. The PG-SGA SF is a widely validated malnutrition screening tool in cancer patients(56). 2) the second part will be completed by the dietician and includes a physical examination, where fat and muscle stores an fluid status are assessed, and assessment of the disease/ condition and metabolic demand. The global PG-SGA generates a subjective category rating for nutritional status: well-nourished (PG-SGA A), suspected malnutrition or moderate malnutrition (PG-SGA B), or severe malnutrition (PG-SGA C). It also provides a numeric score used for triaging nutritional intervention, i.e. to indicate the need for protein andcalorie (en)rich(ed) diet. PG-SGA will be measured at baseline and 1 week before surgery.

- For a first impression of body composition and function, a minimal set of anthropometric measurements are performed. Body weight and height are measured. With these measurements, body frame can be determined, and Body Mass Index (BMI, body weight (kg)/ $(\text{length})^2$ can be calculated. . Measurements will be carried out according to the Anthropometry Procedures Manual of the National Health And Nutrition Survey(57). Body weight and length will be measured at baseline and 1 week before surgery.

- Fat-free mass will be assessed using Bioelectrical impedance analysis (BIA), which is a non-invasive, commonly used method for estimating body composition. BIA allows the determination of the fat-free mass and total body water using appropriate population, age or pathology-specific BIA equations and established procedures(58). BIA will be performed at baseline and 1 week before surgery.

- During the dietetic assessment a 3-day food diary is used to obtain information about patients' food intake. To be able to estimate energy and protein intake, it is sufficient to record food consumption for a period of 3 days, including 2 weekdays and 1 weekend day (59).

- Psychological factors that may affect postoperative outcomes adversely will be identified by the Hospital Anxiety and Depression Scale (HADS). The Hospital Anxiety and Depression Scale (HADS) is developed to identify possible and probable anxiety disorders and depression among patients in nonpsychiatric hospital clinics(60). Physical symptoms, such as dizziness, headaches, insomnia, anergia and fatigue are excluded to prevent noise from somatic disorders. HADS is a validated screening tool for depression and anxiety in different groups of Dutch subjects(61) and is commonly used. HADS will be performed prior to the prehabilitation program to screen whether psychological support during prehabilitation could be of added value.

- Physical activity level will be measured through the Short Questionnaire to Assess Health-enhancing physical activity (SQUASH)(62). This questionnaire contains question on habitual activities with respect to occupation, leisure time, household, transportation means, and other daily activities. SQUASH has been shown to be a fairly reliable and valid tool to gauge the physical activity behavior of the general Dutch adult population(62). SQUASH will be measured at baseline, and at 6 and 12 months after surgery.

- Degree of immunoparalysis; Monocyte HLA-DR (mHLA-DR) expression is chosen based on previous studies showing that 1) mHLA-DR expression is greatly reduced on postoperative day 2, and 2) mHLA-DR expression at this timepoint has predictive value for postoperative infections. mHLA-DR expression will be determined on the following time-points: baseline, prior to surgery, day 2 and week 6 postoperatively. Differences in mHLA-DR expression between 1) prehabilitation and standard of care, and 2) before and after prehabilitation, will be explored. mHLA-DR expression will be measured using Quantibrite methodology (BD Biosciences). This allows for quantification of the absolute number of HLA-DR monoclonal antibodies bound to each monocyte (mAb/cell), which corresponds to the number of HLA-DR molecules expressed on the cell surface. This standardized method is considered the gold standard for HLA-DR analysis and has shown excellent precision, robustness, and predictive value for the development of infections. Blood will be preserved using PROT1 reagent (Smart Tube Inc.), frozen, and analyzed batchwise on a single flow cytometer.

- To compare preoperative health behavior between patients in the control group and patients in the intervention group, a short questionnaire will be developed for each group including questions on physical activity (e.g. number of physiotherapy visits).

8.5 Withdrawal of individual subjects

Subjects can leave the study at any time for any reason if they wish to do so without any consequences. The investigator can decide to withdraw a subject from the study for urgent medical reasons.

8.5.1 Specific criteria for withdrawal (if applicable)

Not applicable

8.6 Replacement of individual subjects after withdrawal

Patients will not be replaced after withdrawal.

8.7 Follow-up of subjects withdrawn from treatment

Regular follow up will be performed for all patients.

8.8 Premature termination of the study

In case of significantly increased incidence of serious side-effects in patients within the intervention group, the study will be ended. Otherwise inclusion will continue. Serious side effects might be exercise related problems.

9. SAFETY REPORTING

9.1 Temporary halt for reasons of subject safety

In accordance to section 10, subsection 4, of the WMO, the sponsor will suspend the study if there is sufficient ground that continuation of the study will jeopardize subject health or safety. The sponsor will notify the accredited METC without undue delay of a temporary halt including the reason for such an action. The study will be suspended pending a further positive decision by the accredited METC. The investigator will take care that all subjects are kept informed.

9.2 AEs, SAEs and SUSARs

9.2.1 Adverse events (AEs)

Adverse events are defined as any undesirable experience occurring to a subject during the study, whether or not considered related to trial procedure. All adverse events reported spontaneously by the subject or observed by the investigator or his staff will be recorded.

9.2.2 Serious adverse events (SAEs)

A serious adverse event is any untoward medical occurrence or effect that

- results in death;
- is life threatening (at the time of the event);
- requires hospitalization or prolongation of existing inpatients' hospitalization;
- results in persistent or significant disability or incapacity;
- is a congenital anomaly or birth defect; or
- any other important medical event that did not result in any of the outcomes listed above due to medical or surgical intervention but could have been based upon appropriate judgement by the investigator.

An elective hospital admission will not be considered as a serious adverse event.

All SAEs will be discussed with participating surgeons/ urologists.

All SAEs will be reported through the web portal *ToetsingOnline* to the accredited METC that approved the protocol, within 15 days after the sponsor has first knowledge of the serious adverse reactions.
SAEs that result in death or are life threatening should be reported expedited. The expedited reporting will occur not later than 7 days after the responsible investigator has first knowledge of the adverse reaction. This is for a preliminary report with another 8 days for completion of the report.

Complications which are directly related to the surgical procedure will not be reported, but will be registered (primary outcome), including:

- wound infection
- rebleeding
- abscess formation
- fascial dehiscence

9.2.3 Suspected unexpected serious adverse reactions (SUSARs)

Not applicable.

9.3 Annual safety report

Not applicable

9.4 Follow-up of adverse events

All AEs will be followed until they have abated, or until a stable situation has been reached. Depending on the event, follow up may require additional tests or medical procedures as indicated, and/or referral to the general physician or a medical specialist.
SAEs need to be reported until the end of the study, as defined in the protocol.

9.5 Data Safety Monitoring Board (DSMB) / Safety Committee

Patients included in the intervention group will be screened for physical fitness prior to the start of prehabilitation. Patients screened at risk will be referred to a cardiologist or pulmonologist to undergo additional tests (e.g. ECG, spirometry, cardiopulmonary exercise test) to assess whether undergoing high intensity training (HIIT) during prehabilitation is safe. Moreover, the content of the prehabilitation program is adapted to each individual participant. Physical measurements and high intensity exercise will be performed under supervision of a 1st line physiotherapist or dietician. If necessary, the program can be adapted throughout the trial over again. Participating in this trial does not alter or delay the treatment in any way. Therefore, the risk of participating

in this trial is negligible and therefore the use of a Data Safety Monitoring Board (DSMB) is not necessary.

10. STATISTICAL ANALYSIS

For this section we consulted Dr. Steven Teerenstra, department of Epidemiology, Radboudumc.

The principle investigator (KvL) has final responsibility with regard to the data. Castor EDC will be used to minimize errors and to ensure traceability.
Data will be analyzed according to a detailed statistical analysis plan, which will be available before the final data freeze to allow for corrections based on data checks and database cleaning.

10.1 Primary study parameter(s)

Data will be analyzed on an intention-to-treat basis. In addition, a per-protocol analysis will be performed.

The primary outcome is postoperative complications. Data will be collected as a continuous variable and calculated through the sum of morbidity and mortality presented on the Clavien-Dindo classification(42). CCI score will be calculated at the 30 day follow up.
This study aims to show a 20% relative reduction in the percentage of Clavien-Dindo score II or higher. To test this, a log-binomial model (generalized linear model with log link and binomial error distribution) will be fitted with clusters (categorical variable), treatment arm, and time (as linear continuous) as fixed effects. Fixed effects for clusters are chosen as the aim is primarily to establish the effect for the Radboudumc. As secondary analyses, both Clavien-Dindo classification and CCI score will be analyzed as semi-continuous scores using generalized linear models with log-link, normal distribution for the errors, and the same fixed effects. Other parametrizations may be investigated if these improve the model fit (e.g, random effects for clusters, other error distributions for instance to account for zero-inflation).

10.2 Secondary study parameter(s)

All secondary data will be described by means plus SD per time point. Generalized linear models as described for the primary parameters will be used. This will be done by using the appropriate link-function (i.e. linear, log, logistic) and error-distribution (i.e, normal, binary). If data are continuous and non-normally distributed, data will be described by means plus IQR per time point. Categorical parameters will be described as number plus percentage per time point. Statistical methods will include t-test and Mann-Whitney U test for continuous normally respectively non-normally distributed parameters at a single time-point post-operatively. Categorical outcomes will be analyzed by using Chi-square tests or (logistic, ordinal or nominal, depending on the definition of the parameter) regression analysis for single time points.

For this section we consulted Dr. Eddy Adang, department of Health Evidence, Radboudumc

The cost-effectiveness analysis will be performed from a healthcare perspective. The aim of the economic evaluation is to measure, value and analyze healthcare costs at a patient's level in patients undergoing usual preoperative care and patients undergoing a multimodal prehabilitation program. The time horizon of the economic evaluation is 12 months after surgery. The analysis will be performed along-side the stepped wedge trial with the primary outcome of the study (postoperative complications).

Standard cost prices for postoperative complications and both prehabilitation programs (PREHAB SHORT and PREHAB LONG) will be determined based on the 'Kostenhandleiding'(63). Data on costs due to postoperative complications will be collected throughout the study in Castor EDC. A multi-level generalized linear model (with gamma distribution and log-link function if data are non-normally distributed) will be used for the cost analysis with chosen fixed effects for time and random effects for clusters.

Additionally, a cost-utility analysis will be performed with quality-adjusted life-years (QALYs) based on the EQ-5D-5L at 6 months after surgery, following the Dutch guidelines of economic evaluation in healthcare(64).

10.3 Other study parameters

Not applicable

10.4 Interim analysis

Recruitment will be monitored. If the sample size per surgical cluster is fewer than expected, more pre-measurements may be retrospectively collected and more post-measurements prospectively. This assessment of recruitment will be done blinded, i.e. without testing the treatment effect.

11. ETHICAL CONSIDERATIONS

11.1 Regulation statement

This study will be conducted according to the principles of the Declaration of Helsinki (59th version, Seoul, October 2008) and other Dutch guidelines, regulations and Acts. Subjects will be coded by a numeric code in order to create an anonymous dataset. Investigators have access to this code and will store the subject identification code list at a separate location from the dataset. Data will be securely stored in the database of the department of surgery of the Radboudumc accessible to the investigators, in accordance with the Dutch Personal Data Protection Act.

11.2 Recruitment and consent

Due to the fact that Dutch guidelines request that oncological patients should be operated within 5 weeks after diagnosis (unless neo-adjuvant therapy is indicated), the timelines for recruitment and informed consent are short to allow enough time for training. Therefore, patients undergoing high impact surgery in the Radboudumc will already be informed about the importance of preoperative condition and nutrition at the referral site. Written information, including the research subject information leaflet and the informed consent form, will be attached to the written invitation for the first outpatient clinic visit in the Radboudumc. Key points of the trial will be repeated during this first outpatient clinic visit. Either the surgeon of case manager will answer additional questions and ask if the patient is willing to participate in the trial by signing the informed consent form. If the patient agrees to participate in this trial, the surgeon or case manager will schedule an appointment (combined with a regular, preoperative outpatient clinic visit) for baseline measurements. Patients can contact an independent doctor (prof. dr. Niels. P. Riksen) for any questions throughout the trial.

11.3 Objection by minors or incapacitated subjects

Not applicable

11.4 Benefits and risks assessment, group relatedness

As mentioned before, it is very likely that patients will benefit from the prehabilitation program intervention since they will be actively working on their physical, mental and nutritional health status. No minors and incapacitated subjects will be included in the study, due to the impossibility to perform all exercises and fulfill all questionnaires. Participating in this trial does not alter or delay the treatment in any way. Side effects might be exercise related problems. Exercise training is not expected to cause any risk to patients. However, since patients are thoroughly screened, we expect to find more pre-existing cardio-pulmonary conditions, for which patients will be referred to either a pulmonologist or cardiologist. The

amount of tests might be perceived as a burden for the patients in both groups, as well as the amount of hospital visits for the prehabilitation group for training and collecting data.

11.5 Compensation for injury

The sponsor/investigator has a liability insurance which is in accordance with article 7 of the WMO.

The sponsor (also) has an insurance which is in accordance with the legal requirements in the Netherlands (Article 7 WMO). This insurance provides cover for damage to research subjects through injury or death caused by the study.
The insurance applies to the damage that becomes apparent during the study or within 4 years after the end of the study.

11.6 Incentives

Participants will not receive a standard financial compensation for participation as an incentive. However, they will receive travel and parking compensation per prehabilitation visit (not the regular outpatient clinic visits) to the hospital.

12. ADMINISTRATIVE ASPECTS, MONITORING AND PUBLICATION

12.1 Handling and storage of data and documents

Data will be managed by an online data management system Castor EDC. Patients receive an unique study number, generated by the data management system. Study number is linked to patient details and stored in a secured file with password. Only the researcher has access to data.

12.2 Monitoring and Quality Assurance

Monitoring will be performed via a risk-based approach, as recommended by the FDA. Although monitoring is legally not required for this study, the sponsor Radboudumc has decided to perform monitoring for all studies that fall under the scope of the WMO-act. Since this study has a minimal risk, monitoring should be performed annually and will partially be done centralized.

12.3 Amendments

Amendments are changes made to the research after a favorable opinion by the accredited METC has been given. All amendments will be submitted to the METC that gave a favorable opinion.

12.4 Annual progress report

The sponsor/investigator will submit a summary of the progress of the trial to the accredited METC once a year. Information will be provided on the date of inclusion of the first subject, numbers of subjects included and numbers of subjects that have completed the trial, serious adverse events/ serious adverse reactions, other problems, and amendments.

12.5 Temporary halt and (prematurely) end of study report

The investigator/sponsor will notify the accredited METC of the end of the study within a period of 8 weeks. The end of the study is defined as the last patient's last visit.

The sponsor will notify the METC immediately of a temporary halt of the study, including the reason of such an action.

In case the study is ended prematurely, the sponsor will notify the accredited METC within 15 days, including the reasons for the premature termination.

Within one year after the end of the study, the investigator/sponsor will submit a final study report with the results of the study, including any publications/abstracts of the study, to the accredited METC.

12.6 Public disclosure and publication policy

The trial is registered in the Netherlands Trial Register under code: NL8699. The research data will be published in relevant scientific journals. Authorship designations will be based on the recommendations of the International Committee of Medical Journal Editors (ICMJE).

13. STRUCTURED RISK ANALYSIS

13.1 Potential issues of concern

Not applicable

13.2 Synthesis

Not applicable

14. APPENDICES

Appendix I: Overview of measurements

OK -4w/OK -8w	OK -1w	OK	OK +1m	OK +6m	OK +12m
Screening<br>*(in intervention group)*	Health behavior questionnaire		Postoperative complications	Physical activity<br>*- SQUASH questionnaire*	Physical activity<br>*- SQUASH questionnaire*
Physical fitness and activity<br>*- submaximal Astrand test*<br>*- indirect 1RM*<br>*- SQUASH questionnaire*	Physical fitness and activity<br>*- submaximal Astrand test*<br>*- indirect 1RM*		Length of stay	Mental health<br>*- SF-36 questionnaire*<br>*- EQ-5D-5L*	Mental health<br>*- SF-36 questionnaire*
Nutritional status<br>*- length, body weight*<br>*- fat-free mass (BIA)*<br>*- 3-day food diary*	Nutritional status<br>*- body weight*<br>*- fat-free mass (BIA)*<br>*- PG-SGA*				
Mental health<br>*- SF-36 questionnaire*	Mental health<br>*- SF-36 questionnaire*				

15. REFERENCES

1. Kirchhoff P, Clavien PA, Hahnloser D. Complications in colorectal surgery: risk factors and preventive strategies. Patient Saf Surg. 2010;4(1):5.
2. McDermott FD, Heeney A, Kelly ME, Steele RJ, Carlson GL, Winter DC. Systematic review of preoperative, intraoperative and postoperative risk factors for colorectal anastomotic leaks. Br J Surg. 2015;102(5):462-79.
3. Govaert JA, Fiocco M, van Dijk WA, Scheffer AC, de Graaf EJ, Tollenaar RA, et al. Costs of complications after colorectal cancer surgery in the Netherlands: Building the business case for hospitals. Eur J Surg Oncol. 2015;41(8):1059-67.
4. Christensen T, Kehlet H. Postoperative fatigue. World J Surg. 1993;17(2):220-5.
5. Kehlet H, Wilmore DW. Evidence-based surgical care and the evolution of fast-track surgery. Ann Surg. 2008;248(2):189-98.
6. Debes C, Aissou M, Beaussier M. [Prehabilitation. Preparing patients for surgery to improve functional recovery and reduce postoperative morbidity]. Ann Fr Anesth Reanim. 2014;33(1):33-40.
7. Li C, Carli F, Lee L, Charlebois P, Stein B, Liberman AS, et al. Impact of a trimodal prehabilitation program on functional recovery after colorectal cancer surgery: a pilot study. Surg Endosc. 2013;27(4):1072-82.
8. Carli F, Charlebois P, Stein B, Feldman L, Zavorsky G, Kim DJ, et al. Randomized clinical trial of prehabilitation in colorectal surgery. Br J Surg. 2010;97(8):1187-97.
9. Demark-Wahnefried W, Aziz NM, Rowland JH, Pinto BM. Riding the crest of the teachable moment: promoting long-term health after the diagnosis of cancer. J Clin Oncol. 2005;23(24):5814-30.
10. Bakens MJ, van der Geest LG, van Putten M, van Laarhoven HW, Creemers GJ, Besselink MG, et al. The use of adjuvant chemotherapy for pancreatic cancer varies widely between hospitals: a nationwide population-based analysis. Cancer Med. 2016;5(10):2825-31.
11. Barberan-Garcia A, Ubre M, Roca J, Lacy AM, Burgos F, Risco R, et al. Personalised Prehabilitation in High-risk Patients Undergoing Elective Major Abdominal Surgery: A Randomized Blinded Controlled Trial. Ann Surg. 2018;267(1):50-6.
12. Carli F, Zavorsky GS. Optimizing functional exercise capacity in the elderly surgical population. Curr Opin Clin Nutr Metab Care. 2005;8(1):23-32.
13. Gustafsson UO, Scott MJ, Schwenk W, Demartines N, Roulin D, Francis N, et al. Guidelines for perioperative care in elective colonic surgery: Enhanced Recovery After Surgery (ERAS((R))) Society recommendations. World J Surg. 2013;37(2):259-84.
14. Schwegler I, von Holzen A, Gutzwiller JP, Schlumpf R, Muhlebach S, Stanga Z. Nutritional risk is a clinical predictor of postoperative mortality and morbidity in surgery for colorectal cancer. Br J Surg. 2010;97(1):92-7.
15. Lohsiriwat V. The influence of preoperative nutritional status on the outcomes of an enhanced recovery after surgery (ERAS) programme for colorectal cancer surgery. Tech Coloproctol. 2014;18(11):1075-80.
16. Tsai S. Importance of lean body mass in the oncologic patient. Nutr Clin Pract. 2012;27(5):593-8.
17. Barbosa LR, Lacerda-Filho A, Barbosa LC. Immediate preoperative nutritional status of patients with colorectal cancer: a warning. Arq Gastroenterol. 2014;51(4):331-6.
18. Deutz NE, Bauer JM, Barazzoni R, Biolo G, Boirie Y, Bosy-Westphal A, et al. Protein intake and exercise for optimal muscle function with aging: recommendations from the ESPEN Expert Group. Clin Nutr. 2014;33(6):929-36.
19. Wall BT, Cermak NM, van Loon LJ. Dietary protein considerations to support active aging. Sports Med. 2014;44 Suppl 2:S185-94.
20. Bauer J, Capra S, Ferguson M. Use of the scored Patient-Generated Subjective Global Assessment (PG-SGA) as a nutrition assessment tool in patients with cancer. Eur J Clin Nutr. 2002;56(8):779-85.
21. Sorensen LT. Wound healing and infection in surgery: the pathophysiological impact of smoking, smoking cessation, and nicotine replacement therapy: a systematic review. Ann Surg. 2012;255(6):1069-79.
22. Sorensen LT, Toft BG, Rygaard J, Ladelund S, Paddon M, James T, et al. Effect of smoking, smoking cessation, and nicotine patch on wound dimension, vitamin C, and systemic

markers of collagen metabolism. Surgery. 2010;148(5):982-90.
23. Thomsen T, Villebro N, Moller AM. Interventions for preoperative smoking cessation. Cochrane Database Syst Rev. 2014(3):Cd002294.
24. Munafo MR, Stevenson J. Anxiety and surgical recovery. Reinterpreting the literature. J Psychosom Res. 2001;51(4):589-96.
25. Kiecolt-Glaser JK, Page GG, Marucha PT, MacCallum RC, Glaser R. Psychological influences on surgical recovery. Perspectives from psychoneuroimmunology. Am Psychol. 1998;53(11):1209-18.
26. Rosenberger PH, Jokl P, Ickovics J. Psychosocial factors and surgical outcomes: an evidence-based literature review. J Am Acad Orthop Surg. 2006;14(7):397-405.
27. Wallace LM. Psychological preparation as a method of reducing the stress of surgery. J Human Stress. 1984;10(2):62-77.
28. Tieland M, Dirks ML, van der Zwaluw N, Verdijk LB, van de Rest O, de Groot LC, et al. Protein supplementation increases muscle mass gain during prolonged resistance-type exercise training in frail elderly people: a randomized, double-blind, placebo-controlled trial. J Am Med Dir Assoc. 2012;13(8):713-9.
29. Pennings B, Koopman R, Beelen M, Senden JM, Saris WH, van Loon LJ. Exercising before protein intake allows for greater use of dietary protein-derived amino acids for de novo muscle protein synthesis in both young and elderly men. Am J Clin Nutr. 2011;93(2):322-31.
30. De Backer IC, Van Breda E, Vreugdenhil A, Nijziel MR, Kester AD, Schep G. High-intensity strength training improves quality of life in cancer survivors. Acta Oncol. 2007;46(8):1143-51.
31. van Waart H, Stuiver MM, van Harten WH, Sonke GS, Aaronson NK. Design of the Physical exercise during Adjuvant Chemotherapy Effectiveness Study (PACES): a randomized controlled trial to evaluate effectiveness and cost-effectiveness of physical exercise in improving physical fitness and reducing fatigue. BMC Cancer. 2010;10:673.
32. van Loon LJ. Role of dietary protein in post-exercise muscle reconditioning. Nestle Nutr Inst Workshop Ser. 2013;75:73-83.
33. Badia JM, Casey AL, Petrosillo N, Hudson PM, Mitchell SA, Crosby C. Impact of surgical site infection on healthcare costs and patient outcomes: a systematic review in six European countries. J Hosp Infect. 2017;96(1):1-15.
34. Alkaaki A, Al-Radi OO, Khoja A, Alnawawi A, Alnawawi A, Maghrabi A, et al. Surgical site infection following abdominal surgery: a prospective cohort study. Can J Surg. 2019;62(2):111-7.
35. Pivot D, Hoch G, Astruc K, Lepelletier D, Lefebvre A, Lucet JC, et al. A systematic review of surgical site infections following day surgery: a frequentist and a Bayesian meta-analysis of prevalence. J Hosp Infect. 2019;101(2):196-209.
36. Leijte GP, Custers H, Gerretsen J, Heijne A, Roth J, Vogl T, et al. Increased Plasma Levels of Danger-Associated Molecular Patterns Are Associated With Immune Suppression and Postoperative Infections in Patients Undergoing Cytoreductive Surgery and Hyperthermic Intraperitoneal Chemotherapy. Front Immunol. 2018;9:663.
37. Timmermans K, Kox M, Vaneker M, van den Berg M, John A, van Laarhoven A, et al. Plasma levels of danger-associated molecular patterns are associated with immune suppression in trauma patients. Intensive Care Med. 2016;42(4):551-61.
38. Warlé M. DEVAR 1-2-3: Surgery induces danger-associated molecule patterns (DAMPs) and innate immune changes in patients undergoing endovascular aneurysm repair - study protocol.
39. NvA/NVvH. Richtlijn: Het preoperatieve traject. 2010.
40. Basse L, Raskov HH, Hjort Jakobsen D, Sonne E, Billesbolle P, Hendel HW, et al. Accelerated postoperative recovery programme after colonic resection improves physical performance, pulmonary function and body composition. Br J Surg. 2002;89(4):446-53.
41. NvA/NVvH. Richtlijn: Het postoperatieve traject. 2010.
42. Dindo D, Demartines N, Clavien PA. Classification of surgical complications: a new proposal with evaluation in a cohort of 6336 patients and results of a survey. Ann Surg. 2004;240(2):205-13.
43. Clavien PA, Barkun J, de Oliveira ML, Vauthey JN, Dindo D, Schulick RD, et al. The Clavien-Dindo classification of surgical complications: five-year experience. Ann Surg. 2009;250(2):187-96.
44. Slankamenac K, Nederlof N, Pessaux P, de Jonge J, Wijnhoven BP, Breitenstein S, et al. The comprehensive complication index: a novel and more sensitive endpoint for assessing

outcome and reducing sample size in randomized controlled trials. Ann Surg. 2014;260(5):757-62; discussion 62-3.
45. Riebe D, Franklin BA, Thompson PD, Garber CE, Whitfield GP, Magal M, et al. Updating ACSM's Recommendations for Exercise Preparticipation Health Screening. Med Sci Sports Exerc. 2015;47(11):2473-9.
46. Magal M, Riebe D. New preparticipation health screening recommendations: what exercise professionals need to know. ACSM's Health & Fitness Journal. 2016;20(3):22-7.
47. Åstrand P-O, Ryhming I. A nomogram for calculation of aerobic capacity (physical fitness) from pulse rate during submaximal work. Journal of applied physiology. 1954;7(2):218-21.
48. Medicine ACoS. ACSM's guidelines for exercise testing and prescription: Lippincott Williams & Wilkins; 2013.
49. Marchese. The essential guide to fitness: for the fitness instructor. 2011:158-9.
50. Mayhew JL, Prinster JL, Ware JS, Zimmer DL, Arabas JR, Bemben MG. Muscular endurance repetitions to predict bench press strength in men of different training levels. J Sports Med Phys Fitness. 1995;35(2):108-13.
51. Gabrielson DK, Scaffidi D, Leung E, Stoyanoff L, Robinson J, Nisenbaum R, et al. Use of an abridged scored Patient-Generated Subjective Global Assessment (abPG-SGA) as a nutritional screening tool for cancer patients in an outpatient setting. Nutr Cancer. 2013;65(2):234-9.
52. Isenring E, Bauer J, Capra S. The scored Patient-generated Subjective Global Assessment (PG-SGA) and its association with quality of life in ambulatory patients receiving radiotherapy. Eur J Clin Nutr. 2003;57(2):305-9.
53. Sealy MJ, Nijholt W, Stuiver MM, van der Berg MM, Roodenburg JL, van der Schans CP, et al. Content validity across methods of malnutrition assessment in patients with cancer is limited. J Clin Epidemiol. 2016;76:125-36.
54. Vigano AL, di Tomasso J, Kilgour RD, Trutschnigg B, Lucar E, Morais JA, et al. The abridged patient-generated subjective global assessment is a useful tool for early detection and characterization of cancer cachexia. J Acad Nutr Diet. 2014;114(7):1088-98.
55. Hakonsen SJ, Pedersen PU, Bath-Hextall F, Kirkpatrick P. Diagnostic test accuracy of nutritional tools used to identify undernutrition in patients with colorectal cancer: a systematic review. JBI Database System Rev Implement Rep. 2015;13(4):141-87.
56. Abbott J, Teleni L, McKavanagh D, Watson J, McCarthy AL, Isenring E. Patient-Generated Subjective Global Assessment Short Form (PG-SGA SF) is a valid screening tool in chemotherapy outpatients. Support Care Cancer. 2016;24(9):3883-7.
57. https://www.cdc.gov/NCHS/data/nhanes/nhanes_07_08/manual_an.pdf.
58. Kyle UG, Bosaeus I, De Lorenzo AD, Deurenberg P, Elia M, Gomez JM, et al. Bioelectrical impedance analysis--part I: review of principles and methods. Clin Nutr. 2004;23(5):1226-43.
59. De Vries JHM, EJ. DB. De voedingsanamnese – Methoden voor voedselconsumptieonderzoek van bevolkingsgroepen en individuen. Informatorium voor Voeding en Diëtetiek, December 2015, Bohn Stafleu van Loghum.
60. Zigmond AS, Snaith RP. The hospital anxiety and depression scale. Acta Psychiatr Scand. 1983;67(6):361-70.
61. Spinhoven P, Ormel J, Sloekers PP, Kempen GI, Speckens AE, Van Hemert AM. A validation study of the Hospital Anxiety and Depression Scale (HADS) in different groups of Dutch subjects. Psychol Med. 1997;27(2):363-70.
62. Wendel-Vos GC, Schuit AJ, Saris WH, Kromhout D. Reproducibility and relative validity of the short questionnaire to assess health-enhancing physical activity. J Clin Epidemiol. 2003;56(12):1163-9.
63. Hakkaart-van Roijen L, Linden Vd, N B, C. , Kanters T, Tan S. Kostenhandleiding: methodologie van kostenonderzoek en referentieprijzen voor economische evaluaties in de gezondheidszorg. In opdracht van Zorginstituut Nederland. Geactualiseerde versie 2015.
64. ZorginstituutNL. Richtlijn voor het uitvoeren van economische evaluaties in de gezondheidszorg. 2016.
