# Supplementary material for: Multimodal prehabilitation (Fit4Surgery) in high-impact surgery to enhance surgical outcomes: Study protocol of F4S PREHAB, a single center stepped wedge trial
Source: PLoS One. 2024 Jul 5;19(7):e0303829. doi: 10.1371/journal.pone.0303829 (PMC11226070; doi:10.1371/journal.pone.0303829)
Supplement: S1 File — (PDF) [file pone.0303829.s003.pdf]

Radboudumc

Radboud universitair medisch centrum
Concernstaf Kwaliteit en Veiligheid
Commissie Mensgebonden Onderzoek
Regio Arnhem-Nijmegen

Postbus 9101, 6500 HB Nijmegen
Huispost 348
Gebouw tandheelkunde
Philips van Leydenlaan 25 (route 348)
T (024) 361 31 54

commissiemensgebondenonderzoek@radboudumc.nl
KvK 41055629/4

628

Radboudumc
Heelkunde
Geert Grooteplein Zuid 10
6525 GA Nijmegen

Ons Kenmerk
PW/CMO 576

Datum
17 december 2020

Titel: Multimodal intensive prehabilitation in high impact surgery
A stepped-wedge cluster randomized trial, from a patient's and hospital-efficiency perspective
Dossiernummer: 2020-6469
NL-nummer: NL73777.091.20

Geachte mevrouw Strijker,

Bijgevoegd treft u aan het positieve oordeel van de CMO Regio Arnhem-Nijmegen over bovengenoemd onderzoek.

Dit betekent dat het onderzoek kan worden uitgevoerd in de centra die in het positieve oordeel worden genoemd nadat de Raden van Bestuur/Directies van die centra daarvoor toestemming hebben verleend.

Indien het voornemen bestaat het onderzoek ook nog in een ander centrum uit te voeren, dan dient aan de CMO Regio Arnhem – Nijmegen een onderzoeksverklaring (zie website www.ccmo.nl) van het betreffende centrum te worden overlegd. De CMO Regio Arnhem-Nijmegen kan vervolgens het positieve oordeel uitbreiden naar het betreffende centrum.

Voorwaarde voor het uitvoeren van een WMO-onderzoek in het Radboudumc is niet alleen het positieve oordeel van de CMO regio Arnhem-Nijmegen (of een andere erkende METC) maar ook de toestemming van de Raad van Bestuur van het Radboudumc. Informatie over de lokale uitvoerbaarheid is gepubliceerd in het Integraal Kwaliteitssysteem voor Wetenschappelijk Mensgebonden Onderzoek.

Ik vertrouw erop u met dit schrijven van dienst te zijn en namens de commissie wens ik u succes met de uitvoering van het onderzoek.

Met vriendelijke groet,
Namens de CMO Regio Arnhem-Nijmegen

Dr. J. Roukema, vicevoorzitter

Kamer van Koophandel - handelsregister 41055629

Radboudumc

BESLUIT
Primaire beoordeling

**NL nummer:**	**NL73777.091.20**	**METC nr.**	**2020-6469**
**Titel onderzoek:**	**Multimodal intensive prehabilitation in high impact surgery A stepped-wedge cluster randomized trial, from a patient's and hospital-efficiency perspective**		

Contactgegevens: Radboudumc, afdeling heelkunde
Verrichter: Radboudumc te Nijmegen

Besluit
De medisch-ethische toetsingscommissie CMO Regio Arnhem-Nijmegen heeft zich, op grond van artikel 2, tweede lid, sub a van de *Wet medisch wetenschappelijk onderzoek met mensen* (WMO), beraden over bovenstaand onderzoeksdossier.

De commissie oordeelt positief over het onderzoeksdossier uit te voeren in het volgende centrum:
- Radboudumc te Nijmegen (hoofdonderzoeker: D. Strijker)

Documenten
Het oordeel is gebaseerd op de documenten die in bijlage 1 zijn vermeld.

Achtergrond
Op 10 juni 2020 is het onderzoeksdossier ter beoordeling bij de commissie ingediend.
Daarna heeft de commissie nadere informatie opgevraagd waarop naar tevredenheid is geantwoord.

Met inachtneming van haar reglement is het onderzoeksdossier besproken in de vergadering van 24 juni 2020; zie bijlage 2 voor de bij de beoordeling betrokken commissieleden.

Overwegingen
De commissie is van oordeel dat aan de voorwaarden in artikel 3 van de WMO is voldaan.

De commissie heeft de inhoud van de onderzoeksverklaring van de deelnemende instelling bekeken. Zij heeft geconstateerd dat is voldaan aan de voorwaarden in artikel 3, onderdeel f, van de WMO. De commissie is van oordeel dat in redelijkheid is voldaan aan het bepaalde in artikel 5 WMO.

De commissie is van oordeel dat het onderzoeksprotocol in een toestemmingsprocedure voorziet die overeenstemt met artikel 6, eerste lid, van de WMO.

De commissie is van mening dat is voldaan aan de voorwaarden in artikel 6, vijfde t/m negende lid, van de WMO. De proefpersonen (en/of degenen die mede/in hun plaats bevoegd zijn tot het geven van toestemming voor deelname aan het onderzoek) worden op gepaste, volledige en begrijpelijke wijze schriftelijk over het onderzoek geïnformeerd en over de mogelijkheid om de toestemming te allen tijde in te trekken.

Kamer van Koophandel - handelsregister 41055629

Verzekeringen
De commissie heeft geconstateerd dat is voldaan aan de verzekeringsplicht. Er is een proefpersonenverzekering afgesloten zoals bepaald in artikel 7, eerste lid, van de WMO en zoals nader uitgewerkt in het Besluit verplichte verzekering bij medisch-wetenschappelijk onderzoek met mensen 2015 (Besluit van 24 november 2014).

Het onderzoek valt onder de proefpersonenverzekering van het Radboudumc.

De commissie heeft geconstateerd dat een aansprakelijkheidsverzekering is afgesloten zoals bepaald in artikel 7, negende lid, van de WMO.

Ten slotte wijst de commissie u op de voorwaarden en verplichtingen die in bijlage 3 zijn vermeld.

Hoogachtend,
Namens de CMO Regio Arnhem-Nijmegen

Dr. J. Roukema, vicevoorzitter

Nijmegen, 17 december 2020

Beroepsprocedure
Tegen dit besluit kan een belanghebbende op grond van artikel 23 van de WMO binnen zes weken na de dag waarop het besluit is bekend gemaakt, administratief beroep instellen bij de Centrale Commissie Mensgebonden Onderzoek (CCMO). Het beroepschrift dient u te adresseren aan CCMO, Postbus 16302, 2500 BH Den Haag.

Kamer van Koophandel - handelsregister 41055629

Radboudumc

Bijlage 1

Documenten:

A	Aanbiedingsbrief d.d. 05 juni 2020<br>Aanbiedingsbrief d.d. 10 november 2020 in reactie op commissievragen d.d. 1 juli 2020<br>Aanbiedingsbrief d.d. 15 november 2020 in reactie op commissievragen d.d. 12 november 2020<br>Aanbiedingsbrief d.d. 14 december 2020 in reactie op commissievragen d.d. 10 december 2020
B	ABR-formulier, versie 02 d.d. 15 november 2020
C	Onderzoeksprotocol versie 9 d.d. 15 november 2020
E	Proefpersoneninformatie, ontvangen d.d. 14 december 2020<br>Toestemmingsformulier, ontvangen d.d. 14 december 2020
F	Vragenlijsten:<br>- ACSM<br>- PG-SGA<br>- HADS<br>- SF-36<br>- SQUASH<br>- Gezondheidsgedrag<br>- Gezondheidsgedrag controlegroep<br>- Gezondheidsgedrag interventiegroep
G	WMO-proefpersonenverzekering van het Radboudumc: van verzekeringsmaatschappij Centramed d.d. januari 2020, datum afgifte RTC CS d.d. 08 juni 2020<br>Bewijs dekking aansprakelijkheid van het Radboudumc: van verzekeringsmaatschappij Centramed d.d. januari 2020
H	CV onafhankelijk arts, prof. dr. N. Riksen<br>CV prof. dr. C. van Laarhoven
I	Onderzoeksverklaring van het Radboudumc te Nijmegen, getekend door afdelingshoofd afdeling Heelkunde C.J.H.M. van Laarhoven inclusief CV lokale hoofdonderzoeker D. Strijker

Kamer van Koophandel - handelsregister 41055629

Radboudumc

Bijlage 2

CMO Regio Arnhem-Nijmegen

Bij de beoordeling betrokken commissieleden

Arts/(vice)voorzitter	J. Roukema
Arts	H. ter Hofstede
Ziekenhuisapotheker/klinisch farmacoloog1	F. Jansman
Arts/klinisch farmacoloog1	B. Schouwenberg
Ethicus	K. ten Cate
Jurist	I. Corte
Methodoloog	R. Donders
Proefpersonenlid	K. Koller
Verpleegkundige	S. Bossmann

Bijlage 3

Voorwaarden en verplichtingen*

- **geen bezwaar bevoegde instantie *[alleen bij geneesmiddelenonderzoek]***:
  er kan pas met het onderzoek worden gestart, wanneer eveneens geen bezwaar wordt gemaakt binnen de voorgeschreven termijn door de bevoegde instantie;
- **geldigheid oordeel**:
  het positieve oordeel verliest zijn geldigheid als de inclusie van de eerste proefpersoon niet heeft plaatsgevonden binnen twee jaar nadat dit besluit is genomen;
- **amendementen**:
  amendementen dienen ter beoordeling aan de CMO Regio Arnhem-Nijmegen te worden voorgelegd;
- **onderzoekscontract**:
  voor aanvang van het onderzoek moet een getekend exemplaar van het goedgekeurde onderzoekscontract bij de CMO Regio Arnhem-Nijmegen ter kennisgeving worden ingediend.
- **multicenter onderzoek**:
  Indien er in het kader van de uitvoering van multicenteronderzoek onderzoekscontracten worden afgesloten, gaat de CMO Regio Arnhem-Nijmegen ervan uit dat de onderzoekscontracten met de overige Nederlandse centra gelijkluidend zijn aan het referentiecontract ten aanzien van de twee onderdelen (criteria voortijdige beëindiging en openbaarmaking onderzoeksresultaten) waarover de CMO Regio Arnhem-Nijmegen oordeelt. Wijkt een lokaal onderzoekscontract op de genoemde punten af van het positief beoordeelde referentiecontract, dan moet dit als amendement ter beoordeling aan de CMO Regio Arnhem-Nijmegen worden voorgelegd.
- **startdatum onderzoek**:
  de CMO Regio Arnhem-Nijmegen dient op de hoogte te worden gesteld van de definitieve startdatum van het onderzoek. Dat is de datum waarop de inclusie van de eerste proefpersoon plaatsvindt;
- **voortgangsrapportage**:
  één jaar na de startdatum, en ieder jaar daaropvolgend, dient de METC op de hoogte te worden gebracht van de voortgang van de studie middels het formulier 'Voortgangsrapportage';
- **geldigheid verzekering**:
  in het geval het verzekeringscertificaat tijdens de voortgang van het onderzoek zijn geldigheid verliest, dient aan de CMO Regio Arnhem-Nijmegen tijdig een afschrift van een nieuw geldig certificaat te worden toegestuurd;
- **melding artikel 10**:
  indien het onderzoek een verloop neemt dat in noemenswaardige mate voor de proefpersoon ongunstiger is dan in het onderzoeksdossier is voorzien, moet daarvan terstond mededeling worden gedaan aan de CMO Regio Arnhem-Nijmegen met een verzoek tot een nader oordeel;
- **melding SAE's**
  SAE's dienen aan de CMO Regio Arnhem-Nijmegen gemeld te worden (raadpleeg de website van de commissie voor de te volgen procedure).
- **melding SUSAR's en jaarlijkse veiligheidsrapportage *[alleen bij geneesmiddelenonderzoek]***:
  SUSAR's en jaarlijkse veiligheidsrapportage dienen aan de CMO Regio Arnhem-Nijmegen gemeld te worden (raadpleeg de website van de commissie voor de te volgen procedure).

- **Advies DSMB** ***[alleen indien een DSMB is ingesteld]***
  indien een advies van de DSMB niet volledig wordt opgevolgd, dient de CMO Regio Arnhem-Nijmegen het advies met toelichting over het niet (volledig) opvolgen van het advies te ontvangen en toestemming te geven voor voortzetting van het onderzoek;
- **melding (voortijdige) beëindiging**:
  (voortijdige) beëindiging van het onderzoek dient, met redenen omkleed, te worden gemeld aan de CMO Regio Arnhem-Nijmegen.
- **eindrapportage**:
  de CMO Regio Arnhem-Nijmegen dient op de hoogte te worden gebracht van de resultaten van het onderzoek middels een eindrapport / (gesubmitte) publicatie.

* Termijnen en overige uitleg ten aanzien van de indiening van de verschillende documenten aan de CMO Regio Arnhem-Nijmegen vindt u op de website van de CCMO bij het standaard onderzoeksdossier en de toelichting daarop en onder stap 4 van het Stappenplan toetsing TC

Kamer van Koophandel - handelsregister 41055629
