# Supplementary material for: Multimodal prehabilitation (Fit4Surgery) in high-impact surgery to enhance surgical outcomes: Study protocol of F4S PREHAB, a single center stepped wedge trial
Source: PLoS One. 2024 Jul 5;19(7):e0303829. doi: 10.1371/journal.pone.0303829 (PMC11226070; doi:10.1371/journal.pone.0303829)
Supplement: S2 File — (PDF) [file pone.0303829.s004.pdf]

Radboudumc

Bijlage 4

Title: Multimodal intensive prehabilitation in high impact surgery
A stepped-wedge cluster randomized trial, from a patient's and hospital-efficiency perspective
Filenumber CMO : 2020-6469

Dear mrs. Strijker,

The medical ethical reviewing committee CMO Regio Arnhem-Nijmegen has reviewed the above-mentioned research file on the grounds of section 2, paragraph 2, sub a of the Medical Research Involving Human Subjects Act (WMO).

The committee has approved the research file on December 17th, 2020.

The decision is based on the documents mentioned in appendix 1 of the original decision written in Dutch.

With kind regards,

On behalf of the CMO Region Arnhem-Nijmegen

Dr. J. Roukema, vice-chairman

Kamer van Koophandel - handelsregister 41055629
