# Supplementary material for: Multimodal prehabilitation (Fit4Surgery) in high-impact surgery to enhance surgical outcomes: Study protocol of F4S PREHAB, a single center stepped wedge trial
Source: PLoS One. 2024 Jul 5;19(7):e0303829. doi: 10.1371/journal.pone.0303829 (PMC11226070; doi:10.1371/journal.pone.0303829)
Supplement: Attachment — Submitted filename: Response to Reviewers.docx [file pone.0303829.s005.docx]

7 March, 2024

PLOS ONE

Subject: Study Protocol Revisions – Response to Reviewers

Dear Editorial Board of PLOS ONE and respected reviewers,

We extend our sincere appreciation for your diligent evaluation and thoughtful feedback on our manuscript. Your thorough consideration is precious to us, and we are grateful for the time and effort you have invested in reviewing our work.

In response to your constructive suggestions and comments, we have carefully addressed each point raised, aiming to enhance the quality and clarity of our research.

We would like to express our gratitude to the entire Editorial Board and reviewers for their valuable insights and guidance throughout this process.

Kind regards,

Luuk D. Drager

MD, MSc, PhD Candidate

Radboud University Medical Center

Department of Surgery, route 618

Postbus 9101, 6500 HB Nijmegen, the Netherlands

Email: luuk.drager@radboudumc.nl

**Reviewer 1**

*Study design is pragmatic rather than an RCT which would yield the highest level of evidence. The authors have argumented well why this is done. It a well designed, clearly described protocol of an important project. I will be keen to see the results.*

Your comment is greatly appreciated. Thank you for taking the time to review our manuscript.

**Reviewer 2**

1. *Sample size. This Section should be rewritten and more details provided. As it stands, it is rather obscure. Please, justify why "a log-binomial model with fixed effects of cluster (specific diagnosis), intervention, and time trend in absence of intervention (as a continuous variable)" is considered. It is rather unclear to me why a log-binomial model should be used in such a context. Similarly, time trend could be non-linear, did you consider the linear term only? Similarly, please clarify what you mean with "assuming a binary distribution that correlates with the estimated number of surgical procedures"; how do you compute correlations involving binary variables? I strongly suggest to write down the proper equations, along with all model's assumptions. The text, as it stands, is not informative to ensure the reproducibility of the method.*

Thank you for your comment. We realize that our initial explanation might have been too brief. Our choice for a log-binomial model is based on the assumption that our intervention, multimodal prehabilitation, will lead to a relative reduction of 20% in complication rates, indicated by the occurrence of a Clavien-Dindo score II or higher. Given the binary nature of the outcome (complication vs. no complication) and our aim to compare relative risks between control and intervention group, we believe the log-binomial model used in our sample size simulation aligns with our research objectives.

You rightly point out the possibility that the time trend could be non-linear. We only assumed the time trend as a linear term in the power calculation as part of the simulation. However, of course, we will further explore this assumption in the statistical analyses of the collected data and will address non-linearity when needed. We changed the text of the manuscript accordingly.

2. *Statistical modelling. Again, it is rather unclear to me why a log-link is considered instead of the canonical logit link; please, make sure that you justify this in detail, aren't you interested in odds-ratios?. Similarly, it is rather weird to assume Gaussian-distributed error terms, as the log-binomial model assumes binomial errors. I strongly suggest to avoid the inclusion of cluster-specific fixed effects (are you using simple dummy variables?), but rather to consider random effects specifications (maybe with random slopes as well, as the effects of the independent variables may be cluster-specific). Please, make clear that the assumptions underlying the modelling will be properly checked to ensure the reliability of statistical inference.*

Our rationale for employing a log-binomial model stems from our objective to estimate risk ratios rather than odds ratios. One important consideration for this is that risk ratios are more accurate than odds ratios when the outcome is prevalent (1). Since we expect our primary outcome (the occurrence of Clavien-Dindo score II or higher) to be relatively common, we believe that using risk ratios for our analysis will be a valid approach.

Regarding the modeling of error terms, there might be some confusion here. We indeed assume binomial-distributed error terms for our log-binomial model, instead of gaussian-distributed error terms. However, our second primary outcome, CCI scores (continuous outcome) requires a different approach and will be modeled using gaussian-distributed error terms. Since this may not have been clearly stated within our statistical analysis paragraph, we revised this in our new manuscript.

We agree with the reviewer’s suggestion that our data may also be modelled using random effects for our surgical indications (clusters). Our suggested approach involves cluster-specific fixed effects, primarily driven as it aligns with our sample size calculation and focus on the Radboudumc effect. However, we acknowledge the importance of assessing the goodness-of-fit for our (proposed) models. Using mixed models is still an option, if this improves the model fit. To make this more clear, we have updated our manuscript to address more explicitly that the underlying assumptions of all our models will be tested. In case of violated assumptions, other parametrizations, such as random effects for clusters, will be used.

3. *Data structure is rather unclear to me. Do you have cross-section, longitudinal or survival-type data? If longitudinal, i.e. repeated measurements, data are available, this data feature must be properly modelled. This is fundamental, as "differences in outcomes between control and intervention group will be tested per single time point" could be misleading, as it ignores the data dynamics.*

Thank you for your comment. Our tertiary data exhibits a longitudinal nature, as measurements are collected at three distinct time points: baseline, 1-2 days prior to surgery, and 3 months post-surgery. Our intention has been to test differences in tertiary outcomes prior to surgery between control and intervention group, as well as three months after surgery. Therefore, we state that “differences in outcomes between control and intervention group will be tested per single time point.” We acknowledge the need for further clarification on this aspect, which we provided in our revised manuscript. Moreover, since the analyses of our tertiary outcomes will be rather explorative, we may also explore the course of these outcomes over time. In case we will do that, the repeated measures in our data will be appropriately accounted for by using linear mixed-effects models.

4. *Economic evaluation. Please, justify why "A multi-level generalized linear model with gamma distribution and log-link function (if data are non-normally distributed) will be used for the cost analysis, with fixed effects for time and random effects for clusters" is considered (here random effects are correctly included in the linear predictor). Costs are known to be far from being Gaussian-distributed, as they are further defined on R+.*

When analyzing healthcare costs, which are often skewed, a generalized model with a gamma distribution is proposed as the most appropriate model (2). We added this reference in our manuscript to substantiate the use of this model distribution as first choice. As in the other analyses, we will also explore distributions and assumptions to ensure the model indeed gives the best fit in the cost analysis.

5. *Goodness of fit of all the proposed models must be carefully checked (along with the underlying assumptions). Please, clarify how do you want to investigate this modelling feature.*

As mentioned before, we underscore the importance of assessing the goodness-of-fit for our (proposed) models in order to ensure validity of statistical inference and choose the best models for our data. In our revised manuscript, we stated more clearly that model assumptions will be tested, and in case of violation, other parametrizations will be used. Moreover, to complement our assessment of goodness-of-fit, we will consult the Aikake information criteria (AIC) as a metric for model selection.

**References**

1.	Knol MJ, Le Cessie S, Algra A, Vandenbroucke JP, Groenwold RH. Overestimation of risk ratios by odds ratios in trials and cohort studies: alternatives to logistic regression. CMAJ. 2012;184(8):895-9.

2.	Malehi AS, Pourmotahari F, Angali KA. Statistical models for the analysis of skewed healthcare cost data: a simulation study. Health Econ Rev. 2015;5:11.
