# Supplementary material for: Multimodal prehabilitation (Fit4Surgery) in high-impact surgery to enhance surgical outcomes: Study protocol of F4S PREHAB, a single center stepped wedge trial
Source: PLoS One. 2024 Jul 5;19(7):e0303829. doi: 10.1371/journal.pone.0303829 (PMC11226070; doi:10.1371/journal.pone.0303829)
Supplement: Attachment — Submitted filename: Response to Reviewers (2).docx [file pone.0303829.s006.docx]

11 April, 2024

PLOS ONE

Subject: Study Protocol Revisions – Response to Reviewers (2)

Dear Editorial Board of PLOS ONE and respected reviewers,

We extend our sincere appreciation for your thorough evaluation and feedback on our manuscript. For this second rebuttal, we sought guidance from Steven Teerenstra, statistician at the Radboudumc renowned for his expertise in stepped wedge design.

To the best of our best knowledge, we have diligently addressed the comments provided by reviewer 2.

We would like to express our profound gratitude to the entire Editorial Board and both reviewers for their valuable insights and guidance, which have contributed to the refinement of our manuscript.

Kind regards,

Luuk D. Drager

MD, MSc, PhD Candidate

Radboud University Medical Center

Department of Surgery, route 618

Postbus 9101, 6500 HB Nijmegen, the Netherlands

Email: luuk.drager@radboudumc.nl

**Reviewer 1**

*“No additional comments. The authors have explained clearly how they revised the manuscript according to the points made by reviewer 2.”*

**Response**

No additional revisions made.

**Reviewer 2**

*“I appreciate the efforts to address my comments. Nevertheless, most of them have been overlooked and minor changes have been added to the main text.

As I mentioned before, models' assumptions must be carefully checked. This is true for the log-binomial model (rarely used in the statistical literature, as risk ratios cannot be calculated in retrospective (case-control) studies, where the total number of exposed people is not available), for which a comparison with more classical approaches is mandatory, but even more for the linear model for which the Gauss Markov assumptions must be checked. Please, provide a detailed residual analysis.

Similarly, the AIC can be used as guidance for model selection, but not to evaluate the goodness-of-fit of the model. Moreover, I am interested in the predictive ability of the log-binomial model. How well does the model recover the observed events?

Random effects modelling should be added and results compared.”*

**Response**

Firstly, we want to underscore one of the selected effect parameters, the risk ratio. We opted for the risk ratio as the effect parameter due to its superior interpretability compared to the odds ratio. Unlike the retrospective (case-control) studies referenced, all our data are prospectively collected as detailed in our manuscript. This also accounts for the data regarding postoperative complications in the historical cohort, since this is extracted from electronic patient files. Furthermore, the risk ratio aligns with the comparison within clusters inherent to the stepped wedge design.

The prespecification of the primary analysis is crucial for mitigating data-dredging risks. Therefore, we will meticulously report the outcome of the prespecified analysis and provide additional analyses as supplementary information. Our selection of the primary analysis was based on simulation studies comparing various models, including random effect modeling with Poisson-error distribution, random effects modeling with binomial-error distribution, and fixed effects modeling with binomial-error distribution. After considering the expected cluster sizes, the fixed effects model with binomial error distribution emerged as the optimal choice, exhibiting superior power and type I error control.

We acknowledge the importance of model assessment, and thus, we intend to evaluate the adequacy of our model by examining whether its predictions systematically over- or underestimate observed data. This evaluation will involve assessing observed versus predicted plots and residual versus predicted plots. Specifically, we will ensure that the distribution of residuals along the predicted axis does not suggest any deviations from normality. Furthermore, we remain open to supplementing our primary analysis model with additional analyses, such as incorporating transformed outcomes, introducing or transforming explanatory variables, or exploring alternative models and link functions to enhance the model fit. We appreciate your suggestion regarding the utilization of a random effect model, and we added this as a supportive analysis, as mentioned in our revised manuscript on page 16 and page 17.

Within models of the same category, the AIC serves as a useful guide for making specific decisions. It is important to note that the log-likelihood of models must be calculated consistently to ensure uniformity; otherwise, the normalizing constant in the AIC would vary. When comparing models across different categories, we will primarily assess the discrepancy between predicted and observed data (prediction error magnitude).

Furthermore, our main focus will be on evaluating the fit at the cluster level. Given that this is a cluster trial, the central inquire revolves around whether the overall outcome of clusters exhibits improvement.
